# Hg^2+^-Promoted Spirolactam Hydrolysis Reaction: A Design Strategy for the Highly Selective Sensing of Hg^2+^ over other Metal Ions in Aqueous Media

**DOI:** 10.3390/s19010128

**Published:** 2019-01-02

**Authors:** Mai Van Bay, Nguyen Khoa Hien, Subin Son, Nguyen Duy Trinh, Nguyen Tien Trung, Pham Cam Nam, Jong Seung Kim, Duong Tuan Quang

**Affiliations:** 1 Department of Chemistry, Hue University, Hue City 84-234, Vietnam; mvbay@ued.udn.vn; 2 Department of Chemistry, The University of Danang-University of Science and Education, Danang City 84-236, Vietnam; 3 Mientrung Institute for Scientific Research, Vietnam Academy of Science and Technology, Hue City 84-234, Vietnam; nguyenkhoahien@yahoo.com; 4 Department of Chemistry, Korea University, Seoul 02841, Korea; babbage91@korea.ac.kr; 5 NTT Hi-Tech Institute, Nguyen Tat Thanh University, Ho Chi Minh City 84-28, Vietnam; ndtrinh@ntt.edu.vn; 6 Laboratory of Computational Chemistry and Modelling, Department of Chemistry, Quy Nhon University, Quy Nhon City 84-256, Vietnam; trung.nguyen@qnu.edu.vn; 7 Department of Chemistry, The University of Danang-University of Science and Technology, Danang City 84-236, Vietnam

**Keywords:** fluorescence, hydrolysis, rhodamine, quantum chemical calculations, mercury

## Abstract

A mercury sensor (*N*-(rhodamine-6G)lactam-ethylenediamine-4-dimethylamino-cinnamaldehyde—RLED) based on the Hg^2+^-promoted hydrolysis reaction has been designed and developed with a combination of theoretical calculations and experimental investigations. The interaction between RLED and Hg^2+^ goes through a fast-initial stage with formation of a 1:1 complex, followed by a slow hydrolysis process. The formation of durable intermediate complexes is due to quite a long hydrolysis reaction time. As a result, RLED can selectively detect Hg^2+^ in the presence of other metal ions, with a detection limit of 0.08 μM for the colorimetric method, and of 0.008 μM with the fluorescent method. In addition, the RLED sensor can work in a solution with a small amount of organic solvent, with a wide pH range from 5 to 10. The time-dependent density functional theory has been used for investigations of the excitation and de-excitation processes in RLED, intermediate complexes, and reaction products, thereby clarifying the changes in the fluorescence intensity before and after the RLED interacts with Hg^2+^ ions.

## 1. Introduction

Heavy metal pollution, especially Hg^2+^, is widespread nowadays and arises from a variety of the natural and anthropogenic sources, for example, oceanic and volcanic emission, mining operation, metallurgy, and other industrial and agricultural activities [1,2]. Organic mercury is formed mainly from the methylation process of inorganic mercury by microorganisms in soil and water [3,4]. At low concentration levels, mercury can cause negative impacts on humans, the environment, animals, and plants [5,6,7]. Because of poisoning by inorganic mercury, humans can suffer from diseases such as digestive system disorders, membranous glomerulonephritis, nephrotic syndrome, spontaneous abortion, and congenital malformation. Meanwhile, poisoning by organic mercury is said to be associated with diseases in humans such as neurological disorders, brain damage, stomatitis, gingivitis, acrodynia, and erythrism [8,9,10].

The development of new fluorescent sensors for mercury ions is always attractive to scientists, in particular fluorescent sensors for selective detection of mercury ions [10,11,12,13,14]. However, as mercury and some other heavy metals have similar electron structures and complexation abilities to ligands, and often appear simultaneously, the fluorescent sensors for mercury ions are often affected by these heavy metals [14]. As a result, there is still a requirement for efficient analytical methods for the highly selective determination of Hg^2+^, especially without the influence of the thiophilic metal ions, such as Cu^2+^ and Ag^+^.

Recently, an attractive design strategy for the highly selective sensing of Hg^2+^ is based on the characteristic reactions of Hg^2+^, such as the reactions of thioureas and amines to produce guanidines in the presence of Hg^2+^ [15,16,17,18,19,20,21], the Hg^2+^-promoted transformation of thiocarbonyl into carbonyl group [22,23], the Hg^2+^-promoted desulfation process to form heterocyclic compounds [24,25,26], the Hg^2+^-promoted deselenization process [27], the thiol elimination reaction from thioether in the presence of Hg^2+^ [28,29], the Hg^2+^-mediated hydration of alkynes to ketones [30,31], and the Hg^2+^-promoted irreversible hydrolysis of the vinyl ether [32] of the isopropenyl acetate [33]. Unfortunately, most of the above characteristic reactions of Hg^2+^ are also based on thiophilic feature. As a result, the reported sensors for Hg^2+^ are also affected by the other thiophilic metal ions. There have been some recent reports that fluorescent sensors based on Hg^2+^-induced catalytic hydrolysis process can selectively detect Hg^2+^ in the presence of other competing metal ions, including Cu^2+^ and Ag^+^ [33,34,35]. However, these fluorescent sensors have still very rarely been reported on until now. Most of them have some limitations, for instance, working in a solution with a large proportion of organic solvents, a narrow pH range, and low sensitivity. In addition, most of the fluorescent sensors were studied in this manner only from experimental investigations [33,34]. So far, there has been still a lack of insight into the nature of the process. This can be clarified by the combination of experimental investigations with quantum chemical calculations, which is still one of the most interesting directions for research to develop new fluorescent sensors and general chemical studies. In particular, this approach also contributes to clarifying the nature of chemical processes [36,37].

**Scheme 1:**
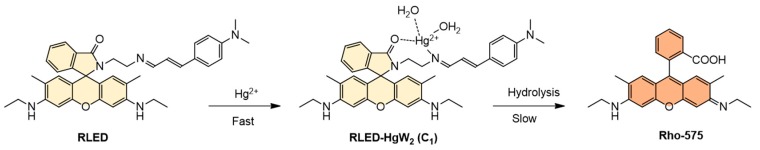
Hg^2+^-induced ring-opening reaction of the spirolactam in (*N*-(rhodamine-6G)lactam-ethylenediamine-4-dimethylamino-cinnamaldehyde—RLED).

In this study, *N*-(rhodamine-6G)lactam-ethylenediamine-4-dimethylamino-cinnamaldehyde (RLED), a new colorimetric and fluorescent sensor for the determination of mercury based on a Hg^2+^-promoted hydrolysis reaction (Scheme 1), was utilized as the recognition event for its high selectivity. This sensor can work in a mild environment, such as at room temperature, water media, and a wide pH range. Furthermore, the Hg^2+^-promoted hydrolysis reaction has been studied using a combination of experimental investigations with quantum chemical calculations, which is a new advancement in this field.

## 2. Materials and Methods

### 2.1. Instruments

A Shimadzu UV-1800 UV-VIS spectrophotometer was used for the UV-VIS absorption spectra. A Shimadzu RF-5301 PC Series fluorescence spectrometer was used for the fluorescence spectra. A Varian instrument was used for the ^1^H-NMR and ^13^C-NMR spectra. A Finnigan 4021C instrument and Daltonics Flex Analysis software were used for the mass spectra.

### 2.2. Reagent

Rhodamine 6G; ethylenediamine; 4-dimethylamino-cinnamaldehyde (DACA); and all of the cationic compounds, such as the chloride (or perchlorate) of Hg^2+^, Cu^2+^, Zn^2+^, Pb^2+^, Cd^2+^, Fe^2+^, Co^2+^, Ni^2+^, Ag^+^, Na^+^, K^+^, Ca^2+^, and Mg^2+^, were purchased from the Aldrich chemical corporation, and were used as received. All of the solvents were HPLC reagent grade solvents without fluorescent impurities, and were purchased from Merck and used as received.

### 2.3. Computational Methodology

The quantum chemical calculations were carried out a using Gaussian 09 program [38] and AIM2000 software [39]. The calculations for the ground state, for example, geometry optimization and single point energy, were carried out using density functional theory (DFT), B3LYP density functional, and the LanL2DZ basis set. The energy values of the reactions such, as the variation of enthalpy (ΔH) and the variation of Gibbs free energy (ΔG), were calculated based on the difference between the total energy of the original compounds and formed compounds. All of the energy values were corrected using for the zero-point energy (ZPE) for the data at 298 K. The calculations for the excited states, for example, the absorption spectra and fluorescence spectra, were performed at the same level of theory, with calculations for the ground state, using the time-dependent density functional theory (TD-DFT) [5,10,37,40,41,42]. The atom in molecule (AIM) theory was applied for analyzing the presence and nature of the bonds in the compounds based on the topological properties of electron density. The stability and characteristics of the bonds were evaluated through the electron density (ρ(r)) and Laplacian ∇^2^(ρ(r)). The bond critical points (BCPs) and the ring critical points (RCPs) were clarified by three eigenvalues (λ_1_ < λ_2_ < λ_3_), which were obtained from the diagonalization of the Hessian of the electron density. They are considered as evidence of the presence of a bond or of a ring structure [10,43,44].

### 2.4. Synthesis

**Scheme 2:**
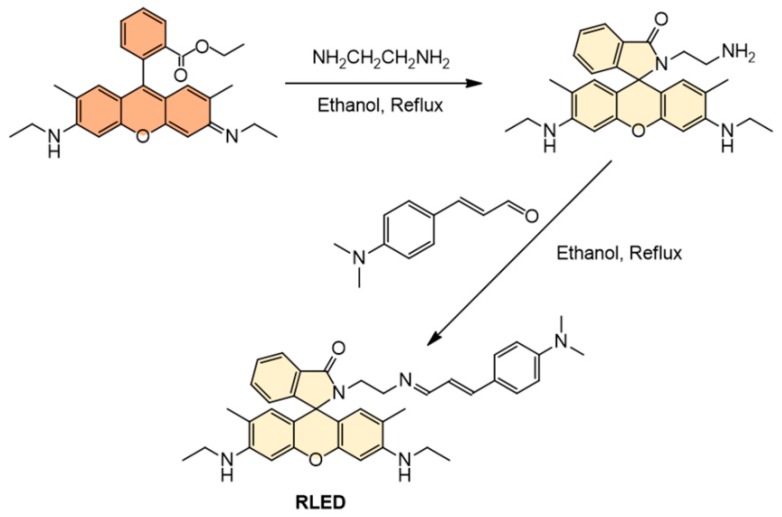
Synthesis scheme for RLED.

The synthetic scheme for RLED is shown in Scheme 2. RLED was prepared from the reaction between rhodamine 6G and ethylenediamine, followed by the reaction with 4-dimethylamino-cinnamaldehyde in a 45% overall yield (see the supplementary data). The structure of RLED was established using ^1^H NMR, ^13^C NMR, and ESI-MS (Figures S1–S5, Supporting Information).

## 3. Results and Discussion

### 3.1. The Experimental Characterization and Application of RLED

**Figure 1:**
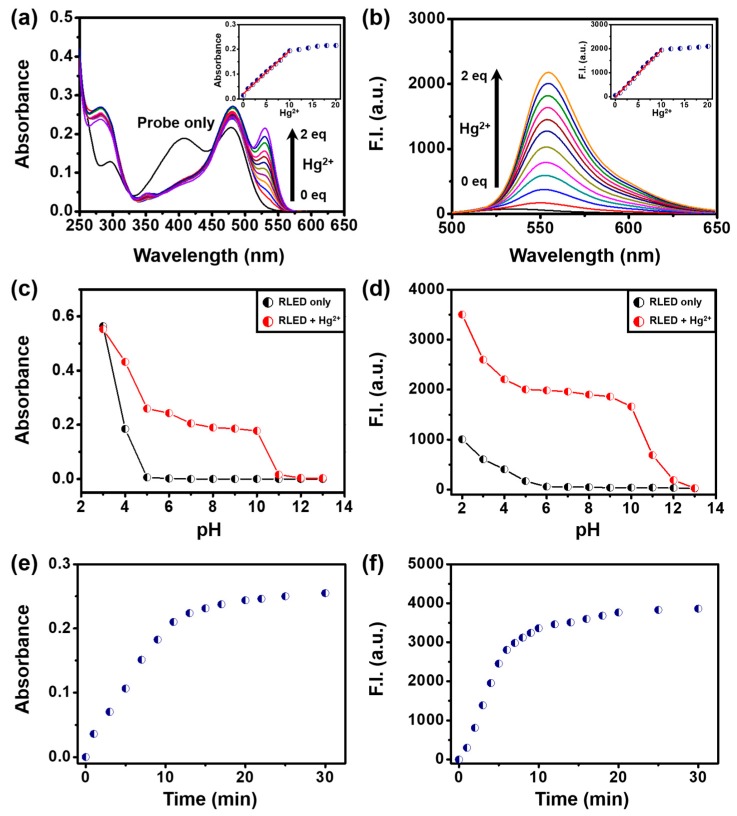
(**a**) Absorbance spectra of RLED (10 μM) in MeOH/HEPES (pH 7.4; 1/9; *v*/*v*) at 25 °C upon increasing the addition of Hg^2+^ (0, 0.1, 0.2, 0.3, 0.4, 0.5, 0.6, 0.7, 0.8, 0.9, 1.0, and 2.0 equivalents) (inset: absorbance at 530 nm vs. the concentration of Hg^2+^ and their linear fitting curve). (**b**) Emission spectra of RLED (10 μM) in MeOH/HEPES (pH 7.4; 1/9; *v*/*v*) at 25 °C upon the gradual addition of Hg^2+^ (0, 0.1, 0.2, 0.3, 0.4, 0.5, 0.6, 0.7, 0.8, 0.9, 1.0, and 2.0 equivalents); excitation wavelength: 530 nm (inset: emission intensity at 558 nm vs. the concentration of Hg^2+^ and their linear fitting curve). All of the spectra were recorded 30 min after the Hg^2+^ addition. (**c**) An absorbance intensity at 530 nm and (**d**) fluorescence intensity at 558 nm of free RLED (10 µM) and RLED +1 equivalent Hg^2+^ in MeOH/H_2_O (1/9; *v*/*v*) with different pH conditions for 30 min. (**e**) Changes of absorbance at 530 nm and (**f**) fluorescence intensity at 558 nm of RLED (10 µM) upon the addition of 1–5 equivalent of Hg^2+^ in MeOH/H_2_O (1/9; *v*/*v*) with the time.

The spectral changes of RLED upon the gradual addition of Hg^2+^ were investigated and are shown in Figure 1. The titration spectra of Hg^2+^ were conducted using 10 µM RLED in methanol/HEPES (pH 7.4; 1/9; *v*/*v*), with the addition of increasing the contents of Hg^2+^ from 1 µM to 20 µM. As shown in Figure 1a, the free RLED displays, the absorption bands peaked at 295, 407, and 480 nm. Upon the addition of the increasing concentration of Hg^2+^, a new absorption band that peaked at 530 nm appeared with a markedly increasing intensity, which induced a color change from colorless to pink. Meanwhile, the obtained results from the fluorescence titration spectra in Figure 1b showed that the free RLED was a non-fluorescent compound. Upon the addition of increasing the concentration of Hg^2+^ to solution of RLED, a new fluorescence band that peaked at 558 nm appeared with increasing intensity, which induced a fluorescence change from colorless to yellow (off–on). The emission intensity at 558 nm induced a 40-fold increase and the absorbance at 530 nm induced a 13-fold increase with addition of 1.0 equivalent of Hg^2+^ to the solution of RLED.

The insets of Figure 1a,b show that there are good linear relationships between the absorbance at 530 nm or fluorescence intensity at 558 nm, and the concentration of Hg^2+^ from 0~1.0 equivalent. Then, upon the addition of concentrations greater than 1 equivalent of Hg^2+^, the absorbance at 530 nm or fluorescence intensity at 558 nm remains unchanged, indicating that the RLED reacts with Hg^2+^ in a 1:1 stoichiometry. With concentrations of Hg^2+^ ranging from 0 to 10 μM, the calibration curve obtained from the linear relationship between the absorbance of RLED and the concentration of Hg^2+^ is ∆A_530nm_ = (0.022 ± 0.002) + (0.017 ± 0.000) × [Hg^2+^], R = 0.997. The calibration curve obtained from the linear relationship between the fluorescence intensity of RLED and the concentration of Hg^2+^ is ∆I_558nm_ = (−34.01 ± 26.79) + (199.17 ± 4.53) × [Hg^2+^], R = 0.995. The detection limits of the colorimetric and fluorescent method for mercury ions are 0.08 and 0.008 μM, respectively (see Figure S9).

From the practical application point of view, the appropriate pH condition of this new sensor was also tested. As shown in Figure 1c,d, when the pH value is reduced from 5 to 2, there is an increase in the absorbance and fluorescence intensity of the free RLED sensor solution. This can be explained by the protonation-caused spirolactam ring-opening in RLED. When the pH value is increased from 5 to 13, the absorbance and fluorescence intensity of the free RLED solution are almost unchanged. However, in the presence of Hg^2+^, there were obvious fluorescence and color (off–on) changes when the pH value was between 5 and 10. In addition, the absorbance and fluorescence intensity of the RLED solution in the presence of Hg^2+^ were also unaffected when the pH value was between 5 and 10. Obviously, these findings suggest that RLED can be used as a colorimetric and fluorescent sensor for the detection of Hg^2+^ with a wide pH span, from 5 to 10.

The reaction time of RLED with Hg^2+^ was also investigated. As shown in Figure 1e,f, the reaction is very rapid in the first 10 min after adding Hg^2+^ to the RLED solution. However, for the best results, the reaction time requires at least 30 min.

**Figure 2:**
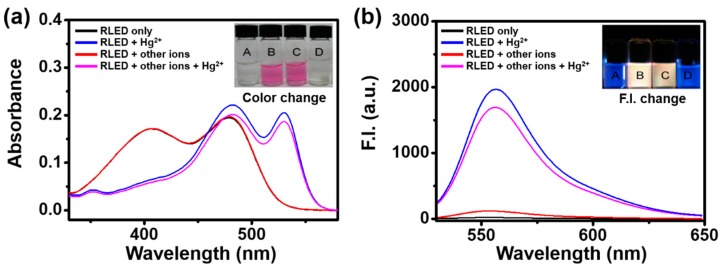
(**a**) Absorbance spectra of RLED (10 μM) in MeOH/HEPES (pH 7.4; 1/9; *v*/*v*) at 25 °C upon the addition of 1 equivalent of Hg^2+^ and other metal ions, including Cu^2+^, Zn^2+^, Pb^2+^, Cd^2+^, Fe^2+^, Co^2+^, Ni^2+^, Ag^+^, Na^+^, K^+^, Ca^2+^, and Mg^2+^ (each 1 equivalent) (inset: color change; A: RLED only; B: RLED + Hg^2+^; C: RLED + X + Hg^2+^; D: RLED + X; X denotes miscellaneous ions, including Cu^2+^, Zn^2+^, Pb^2+^, Cd^2+^, Fe^2+^, Co^2+^, Ni^2+^, Ag^+^, Na^+^, K^+^, Ca^2+^, and Mg^2+^). (**b**) Emission spectra of RLED (10 μM) in MeOH/HEPES (pH 7.4; 1/9; *v*/*v*) at 25 °C upon the addition of 1 equivalent of Hg^2+^ and other metal ions, including Cu^2+^, Zn^2+^, Pb^2+^, Cd^2+^, Fe^2+^, Co^2+^, Ni^2+^, Ag^+^, Na^+^, K^+^, Ca^2+^, and Mg^2+^ (10 equivalent, respectively; excitation wavelength: 530 nm) (inset: fluorescence change). All of the spectra were recorded 30 min after Hg^2+^ addition.

A significant feature of RLED is its high selectivity towards other competitive ions. The variations of the UV-VIS and fluorescence spectra of RLED in the presence of miscellaneous ions, including Cu^2+^, Zn^2+^, Pb^2+^, Cd^2+^, Fe^2+^, Co^2+^, Ni^2+^, Ag^+^, Na^+^, K^+^, Ca^2+^, and Mg^2+^, are displayed in Figure 2a,b. The color change from colorless to pink (off–on) and the fluorescence change from colorless to yellow (off–on) are shown in Figure 2a,b. The competition experiments did not give any significant absorption of the visible region and fluorescence changes. Moreover, in the presence of miscellaneous competitive ions, Hg^2+^ still resulted in similar absorption and fluorescence changes. In addition, the absorbance and fluorescence enhancement resulting from the addition of Hg^2+^ were not influenced by the successive addition of miscellaneous ions. All of these indicate that RLED can be used for the selective recognition of Hg^2+^ in the presence of other competitive metal ions.

### 3.2. The Theoretical Characterization and Application of RLED

**Figure 3:**
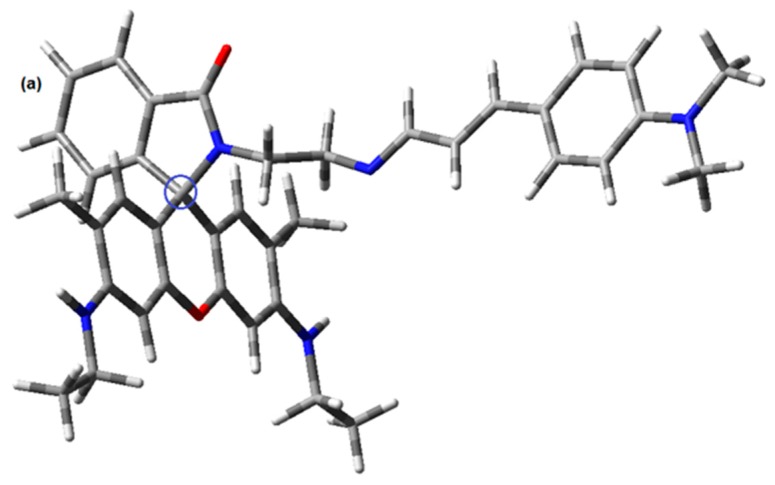

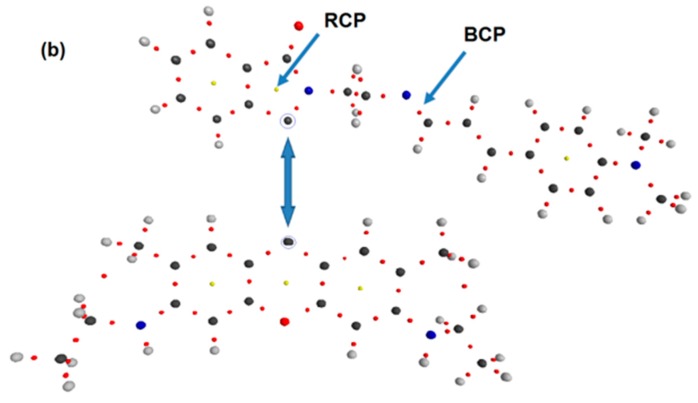
(**a**) The optimized geometry of RLED at the B3LYP/LanL2DZ level of theory. (**b**) The topological properties of RLED at the bond critical points (BCPs) and the ring critical point (RCPs): the red points denote bond critical points (BCPs) and the yellow points denote ring critical points (RCPs).

**Figure 4:**
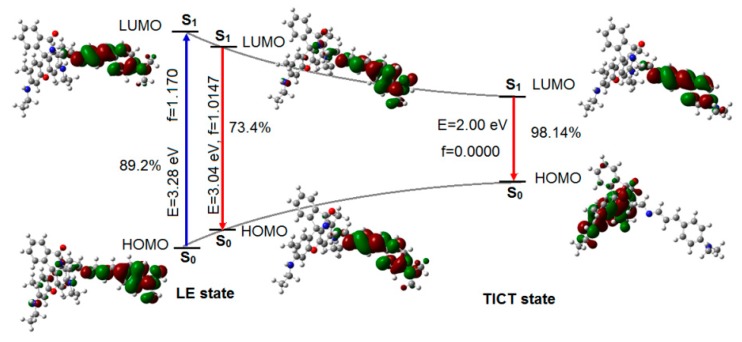
The main transition characteristics (energy (E), oscillator strength (f), percentage contribution, and frontier orbitals) of the excitation and emission processes at the locally excited (LE) state and twisted intermediate charge transfer (TICT) state of RLED at the B3LYP/LanL2DZ level.

The optimized geometry of RLED was carried out at the B3LYP/LanL2DZ level of theory, and is shown in Figure 3 (the Cartesian coordinates given in Table S1). The obtained results show that the RLED molecule is composed of three moieties, including rhodamine, ethylenediamine, and dimethylamino-cinnamaldehyde. The rhodamine moiety is composed of xanthene and phthalimidine subunits, which lie in two planes perpendicular to each other. The phthalimidine subunit in the rhodamine moiety and dimethylamino-cinnamaldehyde moiety are in two planes almost parallel to each other. The topological properties of the BCPs and RCPs obtained from the AIM analysis in Figure 3b also show that there is the presence of a spirolactam ring in the RLED molecule. This makes the π-electron conjugated system in the rhodamine moiety in the RLED molecule broken. Therefore, the rhodamine moiety in the RLED molecule is not a fluorophore. Based on the optimized geometry and the topological properties of RLED, it can be recognized that the DACA moiety in RLED molecule has almost no change compared with the free DACA, therefore it is a fluorophore. However, the obtained experimental results showed that **RLED** did not fluoresce. This was elucidated when studying the absorption and fluorescence spectrum of RLED using the TD-DFT method in Figure 4. The calculated results show that in the ground state (S_0_) and the corresponding S_1_ excited state (also known as the locally excited (LE) state) [45], the dimethylamino group and the phenyl ring of the DACA moiety are almost coplanar. The S_0_ → S_1_ transition (and vice versa) is mainly contributed by the orbital transition from HOMO → LUMO (and vice versa), with a contribution of 89.2% (and 73.4%). In these states, the electron density in HOMO and LUMO is distributed in the DACA moiety. The overlapping of these MOs is large, therefore the above orbital transitions are allowed.

However, because the energy level of S_1_ at the twisted intermediate charge transfer (TICT) state [45], where the dimethylamino group and the phenyl ring of DACA moiety are in two planes perpendicular to each other, is much lower than the energy level of S_1_ at the LE state, the S_1_ excited state transition from the LE state to the TICT state is dominant over the S_1_ → S_0_ transition at the LE state. As a result, S_1_ → S_0_ de-excitation occurs at the TICT state. Unfortunately, the strong charge transfer in the TICT state leads to a complete difference in the localization of the electron density, resulting in the lack of overlapping between HOMO and LUMO. The S_1_ → S_0_ transition at the TICT state is strongly forbidden [46]. These may be the reasons why RLED does not fluoresce.

**Scheme 3:**
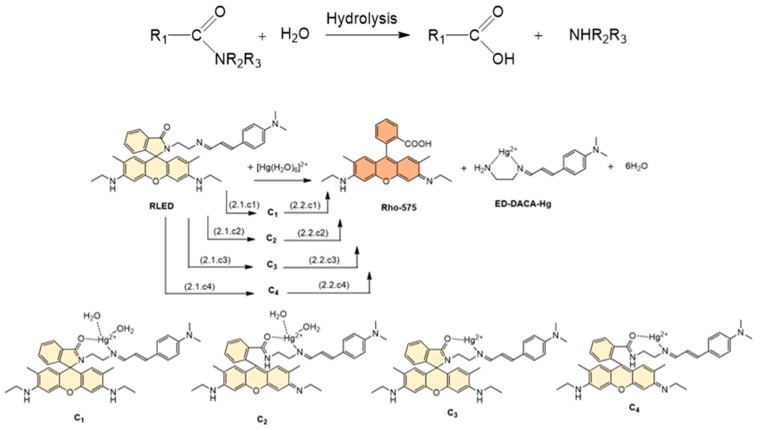
The proposed mechanism for the hydrolysis of RLED.

The reaction between Hg^2+^ and RLED has been elucidated through the quantum chemical calculations. As reported in the experimental results, Hg^2+^ interacts with RLED and induces a fluorescence change of the solution, from colorless to yellow. In addition, one of the obtained experimental products of this reaction is the 2-[3-(ethylamino)-6-ethylimino-2,7-dimethylxanthen-9-yl]benzoic acid (also known as rhodamine 575), with its structure confirmed by ^1^H NMR, ^13^C NMR, and ESI-MS (Figures S6–S8, Supporting Information). These results indicate that this is the hydrolysis reaction of amide (RLED) to form the carboxylic acid and amine in Scheme 3. Generally, the amide hydrolysis reaction is the addition reaction of a nucleophile to the C=O double bond, carried out in either an acidic or basic solution, as same as the ester hydrolysis reaction [47]. Some metal ions, such as Cu^2+^, Ni^2+^, and Zn^2+^, have been known to be capable of promoting an amide hydrolysis reaction, based on their interactions with the O atom in the C=O double bond, which make the carbon atom of the C=O bond become more positive and electrophile [48].

In this case, the experimental results show that the concentration of Hg^2+^ was influential to the hydrolysis reaction of RLED, and Hg^2+^ interacted with RLED in a 1:1 stoichiometry. These results lead to the suggestion that Hg^2+^ reacts with RLED to form an intermediate compound in a 1:1 mole ratio. The calculated results have found four stable configurations (C_1_, C_2_, C_3_, and C_4_) of the 1:1 complexation between Hg^2+^ and RLED in Scheme 3 (the Cartesian coordinates are summarized in Tables S2–S7, Supporting Information).

The variations of enthalpy (*ΔH*) and Gibbs free energy (*ΔG*) of the reactions were calculated and are given in Table 1. For C_1_, C_2_, and C_4_, the complexation process from RLED and Hg^2+^ (reaction (2.1c1), (2.1c2), and (2.1c4)), as well as the decomplexation process to form the final products (reaction (2.2c1), (2.2c2), and (2.2c4)), are thermodynamically favorable. For C_3_, although the reaction forming it from Hg^2+^ and RLED (reaction (2.1c3)) is more thermodynamically favorable than the reactions forming C_1_, C_2_, and C_4_ (reaction (2.1c1), (2.1c2), and (2.1c4)), the decomplexation process from them to form the final products (reaction (2.2c3)) is not thermodynamically favorable. This may be the reason that the hydrolysis reaction time of RLED lasts up to 30 min. These findings indicate that for the sensors for the highly selective detection of Hg^2+^ based on the Hg^2+^-promoted hydrolysis reaction, the design process needs creating a molecular structure of the sensor so that the intermediate complexes with Hg^2+^ are less stable, while the complexes of the byproduct with Hg^2+^ (such as ED-DACA-Hg) are as stable as possible.

**Figure 5:**
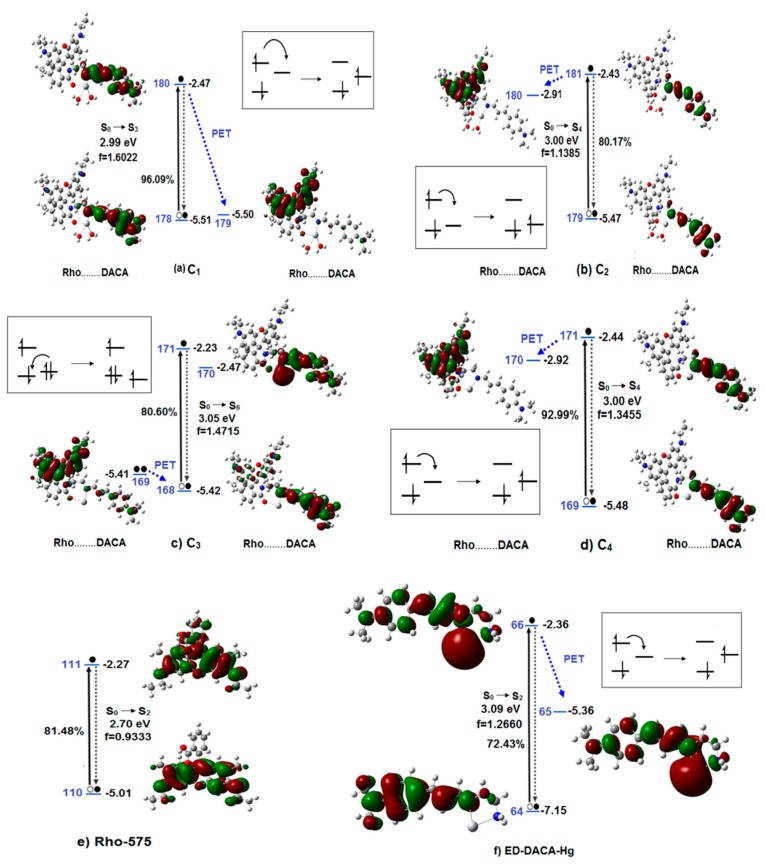
The frontier orbital energy diagram of (**a**) C_1_, (**b**) C_2_, (**c**) C_3_, (**d**) C_4_, (**e**) Rho-575, and (**f**) ED-4-dimethylamino-cinnamaldehyde (DACA)-Hg (the energy levels are relative, not in proportion; summary of the main state transitions with the strongest oscillator strength, and the main orbital transitions with the largest percentage of contribution.

To better understand the nature and to explain the changes in the absorption and fluorescence properties before and after the RLED sensor interacting with Hg^2+^, the time-dependent density functional theory (TD-DFT) was used for calculating the singlet transitions in the compounds studied, at the B3LYP/LanL2DZ level of theory. The calculated results are summarized in Table 2 and Figure 5 (the detailed results are listed in Tables S8–S13 and Figures S10–S15 of supplementary data).

For the C_1_ compound, in all of the transitions from the ground state to excited states, the S_0_ → S_1_ transition is considered as a main state transition, because its oscillator strength (*f*) is the strongest and equals 1.6022. The values of the oscillator strength of the other transition are small and negligible. In all of the orbital transitions of the S_0_ → S_1_ transition, the HOMO-1 → LUMO transition is the main orbital transition, because its percentage contribution is the biggest and equals 96.09%. Because of the presence of HOMO, with its energy level lies between the energy levels of HOMO-1 and LUMO, the photoinduced electron transfer (PET) from the LUMO to HOMO occurs in the excited state in Figure 5a. As a result, the C_1_ complex is a non-fluorescent compound [49].

For the C_2_ compound, the S_0_ → S_2_, S_0_ → S_3_, and S_0_ → S_4_ transitions are the main state transitions, with the oscillator strength of 0.8299, 0.5591, and 1.1385, respectively. The HOMO-1 → LUMO, HOMO-2 → LUMO, and HOMO → LUMO+1 transitions are the main orbital transitions in the above mentioned state transitions, with the percentage contribution of 95.21%, 79.77%, and 80.17%, respectively. For the C_3_ compound, the main state transitions include S_0_ → S_1_ and S_0_ → S_6_ transitions, with the oscillator strength of 0.0689 and 1.4715, respectively. These main state transitions are mainly contributed by the HOMO-1 → LUMO and HOMO-1 → LUMO+1 transitions, with the percentage contribution of 77.33% and 80.00%, respectively. For the C_4_ compound, the main state transition with the strongest oscillator strength (*f* = 1.3455) is the S_0_ → S_4_ transition. It is mainly contributed by the HOMO → LUMO+1, with the percentage contribution of 92.99%. The state transition with the next strong oscillator strength (*f* = 0.8088) is the S_0_ → S_2_ transition. The HOMO → LUMO+1 transition is the orbital transition with the highest percentage contribution in this state transition, with the percentage contribution of 92.80%. The S_0_ → S_3_ transition with the main contribution by the HOMO-2 → LUMO transition (85.61%) is the state transition with the next strong oscillator strength (*f* = 0.4326). Similar to the C_1_ complex, as all of the orbital transitions in the C_2_, C_3_, and C_4_ complex occur between two non-successive MOs, that is, there is always one MO with its energy level is lying between the energy levels of two MOs in each orbital transition, the PET processes occur in the excited state of the complexes in Figure 5b–d [49]. As a result, the C_2_, C_3_, and C_4_ complexes are also non-fluorescent compounds.

Unlike compounds C_1_, C_2_, C_3_, and C_4_, the Rho-575 compound has the S_0_ → S_2_ transition with the strongest oscillator strength (*f* = 0.9333), which it is mainly contributed by a transition between two successive MOs, HOMO and LUMO, with a percentage contribution of 81.48%. Hence, the PET process does not occur in the Rho-575 compound in Figure 5e. This could be the cause that Rho-575 is fluorescent in the experiment [49].

For the ED-DACA-Hg complex, the main state transition (S_0_ → S_2_) with the strongest oscillator strength (*f* = 1.2660) is mainly contributed by a transition between two non-successive MOs, HOMO and LUMO+1, with a percentage contribution of 72.43%. As a result, the PET processes occur in the excited state in Figure 5f, and the ED-DACA-Hg complex is a non-fluorescent compound [49].

## 4. Conclusions

A new sensor, RLED, for the detection of mercury based on the Hg^2+^-promoted hydrolysis reaction has been designed, synthesized, and investigated for application. RLED is able to selectively detect Hg^2+^ over a variety of competitive metal ions, including Cu^2+^, Zn^2+^, Pb^2+^, Cd^2+^, Fe^2+^, Co^2+^, Ni^2+^, Ag^+^, Na^+^, K^+^, Ca^2+^, and Mg^2+^, with the detection limits of 0.08 μM for the colorimetric method, and of 0.008 μM for the fluorescent method. This sensor is able to work in a solution with a small amount of organic solvent (MeOH/water; 1/9; *v*/*v*), and a wide pH range from 5 to 10.

The high selectivity of RLED for Hg^2+^ results from the Hg^2+^-promoted hydrolysis process. This is considered as a design strategy for the highly selective sensing of Hg^2+^ over other metal ions. The nature and mechanism of this process has been elucidated by the quantum chemical calculations based on the previous experimental investigation data. Accordingly, when RLED interacts with Hg^2+^, it first undergoes a fast complexation process followed by a slow hydrolysis process. The formation of durable intermediate complexes may be the reason that the hydrolysis reaction time of RLED lasts up to 30 min. This should be considered when designing new sensors based on this mechanism.

The changes in the fluorescence properties before and after RLED interacts with Hg^2+^ have been clarified by using the time-dependent density functional theory. The S_1_ excited state is transferred from the LE state to the TICT state, followed by the S_1_ → S_0_ non-radiative de-excitation at the TICT state in the form of internal conversion (IC), which causes fluorescence quenching in the RLED sensor. Meanwhile, the orbital transitions between two non-successive MOs lead to the PET process occurring in the excited states, which is thought to be the cause of fluorescence quenching in the C_1_, C_2_, C_3_, C_4_, and ED-DACA-Hg compounds. The orbital transition with a high percentage contribution between two successive MOs leads to the absence of a PET process in the excited states, which causes fluorescence in the Rho-575 compounds.

## Figures and Tables

**Table 1:** The variations of Gibbs free energy (*ΔG*) of the hydrolysis reaction of *N*-(rhodamine-6G)lactam-ethylenediamine-4-dimethylamino-cinnamaldehyde (RLED), and of the intermediate complexation/decomplexation processes in the hydrolysis reaction of RLED (at the B3LYP/LanL2DZ level of theory; 298 K; water; kcal/mol).

Reaction	*∆H^298^*	*∆G^298^*	Reaction	*∆H^298^*	*∆G^298^*
**(2)**	19.46	−34.13			
**(2.1c1)**	−5.88	−27.12	**(2.2c1)**	25.34	−7.02
**(2.1c2)**	−1.74	−24.52	**(2.2c2)**	21.20	−9.61
**(2.1c3)**	−2.95	−47.90	**(2.2c3)**	22.41	13.76
**(2.1c4)**	24.0	−19.21	**(2.2c4)**	−4.63	−14.93

**Table 2:** The calculated excitation energy (E), wavelength (λ), and oscillator strength (*f*) for the low-laying singlet state of C_1_, C_2_, C_3_, C_4_, Rho-575, and ED-4-dimethylamino-cinnamaldehyde (DACA)-Hg at the B3LYP/LanL2DZ level (summary of the main state transitions with the strong oscillator strength, and the main orbital transitions with the large percentage of contribution; in water).

Compound	State	*E* (eV)	λ (nm)	*f*	Contribution of Orbital Transition	
					Composition	Percentage Contribution (%)
**C_1_**	S_0_ → S_3_	2.99	414.4	1.6022	HOMO-1 → LUMO	96.09
**C_2_**	S_0_ → S_2_	2.74	452.5	0.8299	HOMO-1 → LUMO	95.21
	S_0_ → S_3_	2.89	429.0	0.5591	HOMO-2 → LUMO	79.77
	S_0_ → S_4_	3.01	412.4	1.1385	HOMO → LUMO+1	80.17
**C_3_**	S_0_ → S_1_	2.49	498.1	0.0689	HOMO-1 → LUMO	77.33
	S_0_ → S_6_	3.05	407.2	1.4715	HOMO-1 → LUMO+1	80.0
**C_4_**	S_0_ → S_2_	2.74	453.1	0.8088	HOMO-1 → LUMO	92.80
	S_0_ → S_3_	2.81	441.9	0.4326	HOMO-2 → LUMO	85.61
	S_0_ → S_4_	3.00	413.8	1.3455	HOMO → LUMO+1	92.99
**Rho-575**	S_0_ → S_2_	2.70	460.0	0.9333	HOMO → LUMO	81.48
**ED-DACA-Hg**	S_0_ → S_1_	2.61	475.1	0.1411	HOMO → LUMO	72.81
	S_0_ → S_2_	3.09	401.1	1.2660	HOMO → LUMO+1	72.43

## Supplementary Materials

The following are available online at http://www.mdpi.com/1424-8220/19/1/128/s1: Figure S1: ^1^H NMR spectra of *N*-(rhodamine-6G)lactam-ethylenediamine; Figure S2: TOF-Mass spectrum of *N*-(rhodamine-6G)lactam-ethylenediamine; Figure S3: ^1^H NMR spectra of RLED; Figure S4: ^13^C NMR spectra of RLED; Figure S5: ESI Mass spectrum of RLED; Figure S6: ^1^H NMR spectra of Rho-575; Figure S7: ^13^C NMR spectra of Rho-575; Figure S8: ESI Mass spectrum of Rho-575; Figure S9: (**a**) Absorbance at 530 nm vs. the concentration of Hg^2+^ ions and their linear fitting curve. (**b**) Emission intensity at 558 nm vs. the concentration of Hg^2+^ ions and their linear fitting curve. All of the spectra were recorded at 30 min after Hg^2+^ addition; RLED (10 μM) in MeOH/HEPES (pH 7.4; 1/9; *v*/*v*) at 25 °C; Table S1: XYZ coordinates for calculated optimized geometry of RLED. Table S2: XYZ coordinates for calculated optimized geometry of C_1_. Table S3: XYZ coordinates for calculated optimized geometry of C_1_. Table S4: XYZ coordinates for calculated optimized geometry of C_3_. Table S5: XYZ coordinates for calculated optimized geometry of C_4_. Table S6: XYZ coordinates for calculated optimized geometry of Rho-575. Table S7: XYZ coordinates for calculated optimized geometry of ED-DACA-Hg. Table S8: The calculated excitation energy (E), wavelength (λ), and oscillator strength (f) for low-laying singlet state of C_1_ at the B3LYP/LanL2DZ level (in water). Table S9: The calculated excitation energy (E), wavelength (λ), and oscillator strength (f) for low-laying singlet state of C_2_ at the B3LYP/LanL2DZ level (in water). Table S10: The calculated excitation energy (E), wavelength (λ), and oscillator strength (f) for low-laying singlet state of C_3_ at the B3LYP/LanL2DZ level (in water). Table S11: The calculated excitation energy (E), wavelength (λ), and oscillator strength (f) for low-laying singlet state of C_4_ at the B3LYP/LanL2DZ level (in water). Table S12: The calculated excitation energy (E), wavelength (λ), and oscillator strength (f) for low-laying singlet state of Rho-575 at the B3LYP/LanL2DZ level (in water). Table S13: The calculated excitation energy (E), wavelength (λ), and oscillator strength (f) for low-laying singlet state of ED-DACA-Hg at the B3LYP/LanL2DZ level (in water). Figure S10: The frontier orbital energy diagram of C_1_ (the energy levels are relative, not in proportion). Figure S11: The frontier orbital energy diagram of C_2_ (the energy levels are relative, not in proportion). Figure S12: The frontier orbital energy diagram of C_3_ (the energy levels are relative, not in proportion). Figure S13: The frontier orbital energy diagram of C_4_ (the energy levels are relative, not in proportion). Figure S14: The frontier orbital energy diagram of Rho-575 (the energy levels are relative, not in proportion). Figure S15: The frontier orbital energy diagram of ED-DACA-Hg (the energy levels are relative, not in proportion).

## References

[1] Chen X.Q., Nam S.W., Jou M.J., Kim Y., Kim S.J., Park S., Yoon J. (2008). Hg^2+^ Selective Fluorescent and Colorimetric Sensor: Its Crystal Structure and Application to Bioimaging. Org. Lett..

[2] Raikwar M.K., Kumar P., Singh M., Singh A. (2008). Toxic effect of heavy metals in livestock health. Vet. World.

[3] Flora S.J.S., Mittal M., Mehta A. (2008). Heavy metal induced oxidative stress & its possible reversal by chelation therapy. Indian J. Med. Res..

[4] Hien N.K., Quy P.T., Trung N.T., Vien V., Van Khanh D., Nhung N.T.A., Quang D.T. (2014). A Dansyl–Diethylenetriamine–Thiourea Conjugate as a Fluorescent Chemodosimeter for Hg^2+^ Ions in Water Media. Chem. Lett..

[5] Nhan D.T., Nhung N.T.A., Vien V., Trung N.T., Cuong N.D., Bao N.C., Huong D.Q., Hien N.K., Quang D.T. (2017). A Benzothiazolium-derived Colorimetric and Fluorescent Chemosensor for Detection of Hg^2+^ Ions. Chem. Lett..

[6] Quy P.T., Hien N.K., Bao N.C., Nhan D.T., Khanh D.V., Nhung N.T.A., Tung T.Q., Luyen N.D., Quang D.T. (2015). A new rhodamine-based fluorescent chemodosimeter for mercuric ions in water media. Luminescence.

[7] Lee M.H., Wu J.S., Lee J.W., Jung J.H., Kim J.S. (2007). Highly sensitive and selective chemosensor for Hg^2+^ based on the rhodamine fluorophore. Org. Lett..

[8] Duruibe J.O., Ogwuegbu M., Egwurugwu J. (2007). Heavy metal pollution and human biotoxic effects. Int. J. Phys. Sci..

[9] Ibrahim D., Froberg B., Wolf A., Rusyniak D.E. (2006). Heavy metal poisoning: Clinical presentations and pathophysiology. Clin. Lab. Med..

[10] Hien N.K., Bao N.C., Nhung N.T.A., Trung N.T., Nam P.C., Duong T., Kim J.S., Quang D.T. (2015). A highly sensitive fluorescent chemosensor for simultaneous determination of Ag (I), Hg (II), and Cu (II) ions: Design, synthesis, characterization and application. Dyes Pigments.

[11] Hou J.T., Ren W.X., Li K., Seo J., Sharma A., Yu X.Q., Kim J.S. (2017). Fluorescent bioimaging of pH: From design to applications. Chem. Soc. Rev..

[12] Quang D.T., Hop N.V., Luyen N.D., Thu H.P., Oanh D.Y., Hien N.K., Hieu N.V., Lee M.H., Kim J.S. (2013). A new fluorescent chemosensor for Hg^2+^ in aqueous solution. Luminescence.

[13] Jana A., Kim J.S., Jung H.S., Bharadwaj P.K. (2009). A cryptand based chemodosimetric probe for naked-eye detection of mercury(II) ion in aqueous medium and its application in live cell imaging. Chem. Commun..

[14] Hu J.M., Wu T., Zhang G.Q., Liu S.Y. (2012). Highly Selective Fluorescence Sensing of Mercury Ions over a Broad Concentration Range Based on Mixed Polymeric Micelles. Macromolecules.

[15] Zou Q., Zou L., Tian H. (2011). Detection and adsorption of Hg^2+^ by new mesoporous silica and membrane material grafted with a chemodosimeter. J. Mater. Chem..

[16] Sumiya S., Sugii T., Shiraishi Y., Hirai T. (2011). A benzoxadiazole-thiourea conjugate as a fluorescent chemodosimeter for Hg(II) in aqueous media. J. Photochem. Photobiol. A Chem..

[17] Lu Z.J., Wang P.N., Zhang Y., Chen J.Y., Zhen S., Leng B., Tian H. (2007). Tracking of mercury ions in living cells with a fluorescent chemodosimeter under single- or two-photon excitation. Anal. Chim. Acta.

[18] Lee M.H., Lee S.W., Kim S.H., Kang C., Kim J.S. (2009). Nanomolar Hg(II) Detection Using Nile Blue Chemodosimeter in Biological Media. Org. Lett..

[19] Guo Z., Zhu W.H., Zhu M.M., Wu X.M., Tian H. (2010). Near-Infrared Cell-Permeable Hg^2+^-Selective Ratiometric Fluorescent Chemodosimeters and Fast Indicator Paper for MeHg^+^ Based on Tricarbocyanines. Chem. Eur. J..

[20] Lee M.H., Kim J.S., Sessler J.L. (2015). Small molecule-based ratiometric fluorescence probes for cations, anions, and biomolecules. Chem. Soc. Rev..

[21] Kim H.N., Ren W.X., Kim J.S., Yoon J. (2012). Fluorescent and colorimetric sensors for detection of lead, cadmium, and mercury ions. Chem. Soc. Rev..

[22] Yang Y.M., Zhao Q., Feng W., Li F.Y. (2013). Luminescent Chemodosimeters for Bioimaging. Chem. Rev..

[23] Lee J.W., Jung H.S., Kwon P.S., Kim J.W., Bartsch R.A., Kim Y., Kim S.J., Kim J.S. (2008). Chromofluorescent indicator for intracellular Zn^2+^/Hg^2+^ dynamic exchange. Org. Lett..

[24] Zhao Y.G., Lin Z.H., He C., Wu H.M., Duan C.Y. (2006). A “turn-on” fluorescent sensor for selective Hg(II) detection in aqueous media based on metal-induced dye formation. Inorg. Chem..

[25] Kim H.N., Lee M.H., Kim H.J., Kim J.S., Yoon J. (2008). A new trend in rhodamine-based chemosensors: application of spirolactam ring-opening to sensing ions. Chem. Soc. Rev..

[26] Jiang W., Wang W. (2009). A selective and sensitive “turn-on” fluorescent chemodosimeter for Hg^2+^ in aqueous media via Hg^2+^ promoted facile desulfurization-lactonization reaction. Chem. Commun..

[27] Samb I., Bell J., Toullec P.Y., Michelet V., Leray I. (2011). Fluorescent Phosphane Selenide as Efficient Mercury *Chemodosimeter*. Org. Lett..

[28] Cheng X.H., Li Q.Q., Qin J.G., Li Z. (2010). A New Approach to Design Ratiometric Fluorescent Probe for Mercury(II) Based on the Hg^2+^-Promoted Deprotection of Thioacetals. ACS Appl. Mater. Interfaces.

[29] Cheng X.H., Li S., Zhong A.S., Qin J.G., Li Z. (2011). New fluorescent probes for mercury(II) with simple structure. Sens. Actuators B Chem..

[30] Song F.L., Watanabe S., Floreancig P.E., Koide K. (2008). Oxidation-Resistant Fluorogenic Probe for Mercury Based on Alkyne Oxymercuration. J. Am. Chem. Soc..

[31] Ando S., Koide K. (2011). Development and Applications of Fluorogenic Probes for Mercury(II) Based on Vinyl Ether Oxymercuration. J. Am. Chem. Soc..

[32] Lee H., Kim H.J. (2011). Ratiometric fluorescence chemodosimeter for mercuric ions through the Hg(II)-mediated propargyl amide to oxazole transformation. Tetrahedron Lett..

[33] Du J.J., Fan J.L., Peng X.J., Sun P.P., Wang J.Y., Li H.L., Sun S.G. (2010). A New Fluorescent Chemodosimeter for Hg^2+^: Selectivity, Sensitivity, and Resistance to Cys and GSH. Org. Lett..

[34] Gao Y.L., Zhang C., Peng S.W., Chen H.Y. (2017). A fluorescent and colorimetric probe enables simultaneous differential detection of Hg^2+^ and Cu^2+^ by two different mechanisms. Sens. Actuators B Chem..

[35] Manna S., Karmakar P., Maiti K., Ali S.S., Mandal D., Mahapatra A.K. (2017). A reactive primary fluorescence switch-on sensor for Hg^2+^ and the generated fluorophore as secondary recognition receptor toward Cu^2+^ in aqueous acetonitrile solution. J. Photochem. Photobiol. A Chem..

[36] Becke A.D. (1996). Density-functional thermochemistry. IV. A new dynamical correlation functional and implications for exact-exchange mixing. J. Chem. Phys..

[37] Becke A.D. (1993). Density-functional thermochemistry. III. The role of exact exchange. J. Chem. Phys..

[38] Frisch M.J., Trucks G.W., Schlegel H.B., Scuseria G.E., Robb M.A., Cheeseman J.R., Scalmani G., Barone V., Petersson G.A., Nakatsuji H. (2016). Gaussian 16.

[39] Biegler-König F. (2000). Update of the AIM2000-Program for atoms in molecules. J. Comput. Chem..

[40] Stratmann R.E., Scuseria G.E., Frisch M.J. (1998). An efficient implementation of time-dependent density-functional theory for the calculation of excitation energies of large molecules. J. Chem. Phys..

[41] Nhan D.T., Hien N.K., Duc H.V., Nhung N.T.A., Trung N.T., Van D.U., Shin W.S., Kim J.S., Quang D.T. (2016). A hemicyanine complex for the detection of thiol biomolecules by fluorescence. Dyes Pigments.

[42] Lee C.T., Yang W.T., Parr R.G. (1988). Development of the Colle-Salvetti Correlation-Energy Formula into a Functional of the Electron-Density. Phys. Rev. B.

[43] Koch U., Popelier P.L.A. (1995). Characterization of C-H-O Hydrogen-Bonds on the Basis of the Charge-Density. J. Phys. Chem..

[44] Bader R.F.W. (1991). A Quantum-Theory of Molecular-Structure and Its Applications. Chem. Rev..

[45] Valeur B. (2001). Molecular Fluorescence: Principles and Applications.

[46] Hien N.K., Nhan D.T., Kim W.Y., Van Bay M., Nam P.C., Van D.U., Lim I.T., Kim J.S., Quang D.T. (2018). Exceptional case of Kasha’s rule: Emission from higher-lying singlet electron excited states into ground states in coumarin-based biothiol sensing. Dyes Pigments.

[47] Roberts J.D., Caserio M.C. (1977). Basic Principles of Organic Chemistry.

[48] Sayre L.M., Jacobson A.R. (1986). Metal-Ion Catalysis of Amide Hydrolysis. Abstr. Pap. Am. Chem. Soc..

[49] de Silva A.P., Moody T.S., Wright G.D. (2009). Fluorescent PET (Photoinduced Electron Transfer) sensors as potent analytical tools. Analyst.

