# Supplementary material for: Hg2+-Promoted Spirolactam Hydrolysis Reaction: A Design Strategy for the Highly Selective Sensing of Hg2+ over other Metal Ions in Aqueous Media
Source: Sensors (Basel). 2019 Jan 2;19(1):128. doi: 10.3390/s19010128 (PMC6338984; doi:10.3390/s19010128)
Supplement: Supplementary file 1 [file sensors-19-00128-s001.pdf]

Supporting Information

Hg^{2+}-promoted spirolactam hydrolysis reaction: a design strategy for highly selective sensing of Hg^{2+} over other metal ions in aqueous media

Mai Van Bay [1,2,†], Nguyen Khoa Hien [3,†], Subin Son [4,†], Nguyen Duy Trinh [5], Nguyen Tien Trung [6], Pham Cam Nam [7,*], Jong Seung Kim [4,*] and Duong Tuan Quang [1,*]

[1] Department of Chemistry, Hue University, Hue City 84-234, Vietnam; mvbay@ued.udn.vn
[2] The University of Danang, University of Science and Education, Danang City 84-236, Vietnam
[3] Mientrung Institute for Scientific Research, Vietnam Academy of Science and Technology, Hue City 84-234, Vietnam; nguyenkhoahien@yahoo.com
[4] Department of Chemistry, Korea University, Seoul 02841, Korea; babbage91@korea.ac.kr
[5] NTT Hi-Tech Institute, Nguyen Tat Thanh University, Ho Chi Minh City 84-28, Vietnam; ndtrinh@ntt.edu.vn
[6] Laboratory of Computational Chemistry and Modelling, Department of Chemistry, Quy Nhon University, Quy Nhon City 84-256, Vietnam; trung.nguyen@qnu.edu.vn
[7] The University of Danang, University of Science and Technology, Danang City 84-236, Vietnam
† These authors contributed equally to the work
* Correspondence: camnamp@yahoo.com (P.C.N.); jongskim@korea.ac.kr (J.S.K.); duongtuanquang@dhsphue.edu.vn (D.T.Q.)

1. Synthesis

* Synthesis of *N*-(rhodamine-6G)lactam-ethylenediamine

Rhodamine 6G (480 mg, 1 mmol), ethylenediamine (0.67 mL, 10 mmol) and 20 mL of ethanol were added to a 100 mL flask. The reaction mixture was refluxed for 4 hours till the fluorescence of the reaction vanished. The reaction was cooled to room temperature, and the precipitate was collected and washed with cold ethanol for several times. Crude product was purified by recrystallization from acetonitrile to give 370 mg of ***N*-(rhodamine-6G)lactam-ethylenediamine** (white solid) in 80.1% yield. ^{1}H-NMR ($CDCl_3$): δ 7.95 (d, 1H), 7.47 (t, 2H), 7.05 (d, 1H), 6.34 (s, 2H), 6.23 (s, 2H), 3.50 (t, 2H), 3.24 (t, 4H), 2.39 (t, 2H), 1.90 (s, 6H), 1.36 (t, 6H). ESI-MS (M+H$^+$): m/z=457.

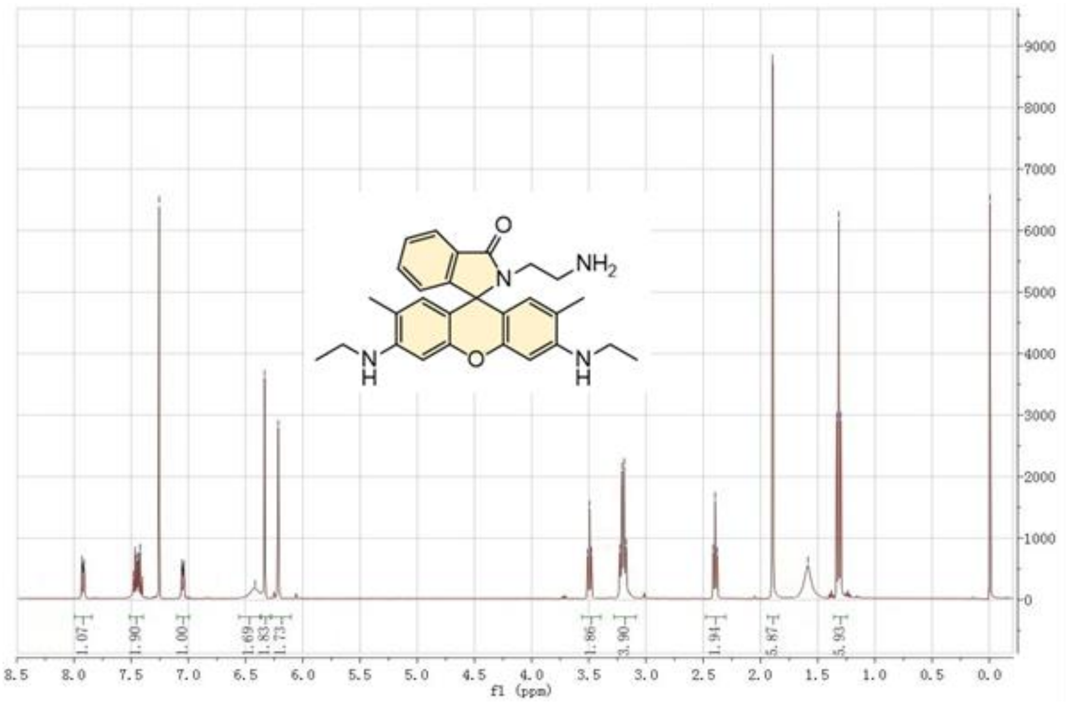

Figure S1. ^{1}H NMR spectra of N-(rhodamine-6G)lactam-ethylenediamine.

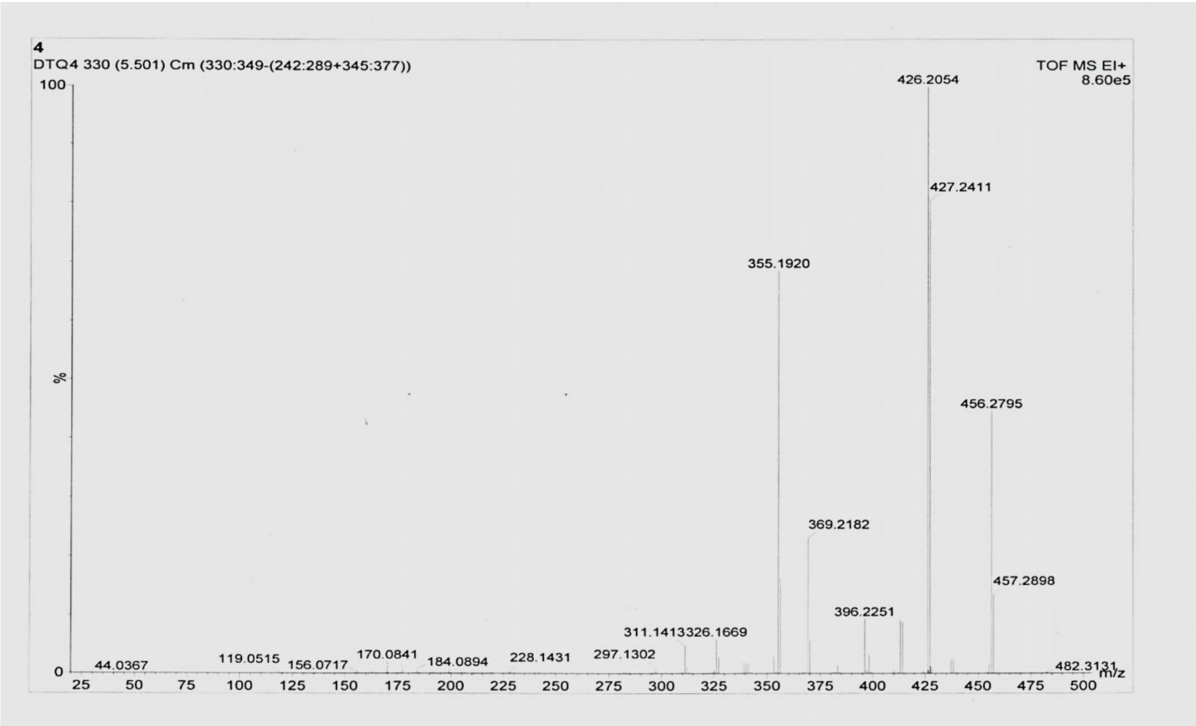

Figure S2. TOF-Mass spectrum of N-(rhodamine-6G)lactam-ethylenediamine.

* Synthesis of RLED

A portion of *N*-(rhodamine-6G)lactam-ethylenediamine (456 mg, 1.0 mmol) and 4-dimethylamino-cinnamaldehyde (350 mg, 2 mmol) were combined in 50 mL of absolute ethanol. The reaction mixture was refluxed for 8 h under N_2 atmosphere and stirred for another 2 h at room temperature to precipitate the white solid. The solid was filtrated, washed with ethanol three times. Crude product was purified by recrystallization from acetonitrile to give 338.2 mg of **RLED** in 55.5%

yield. ^{1}H-NMR ($CDCl_3$) δ 7.94-7.92 (d, 1H), 7.70-7.68 (d, 1H), 7.43-7.40 (m, 2H), 7.30-7.28 (d, 2H), 7.05-7.03 (d, 2H), 6.75-6.71 (d, 1H), 6.66-6.64 (d, 1H), 6.48 (m, 1H), 6.32 (s, 2H), 6.22 (s, 2H), 3.49-3.45 (t, 2H), 3.38-3.45 (t, 2H), 6.27-3.23 (t, 2H), 3.22-3.19 (t, 4H), 2.98 (s, 6H), 1.88 (s, 6H), 1.59 (s, 6H). ^{13}C-NMR ($CDCl_3$) δ: 168.46, 164.59, 154.07, 152.03, 151.16, 147.59, 142.00, 132.45, 131.50, 128.77, 128.72, 128.06, 124.30, 124.10, 123.99, 122.93, 118.01, 112.30, 106.52, 100.20, 96.95, 65.35, 58.74, 41.73, 40.31, 38.53, 16.70, 14.93 ppm. ESI-MS ($M+H^+$): m/z=614.3466.

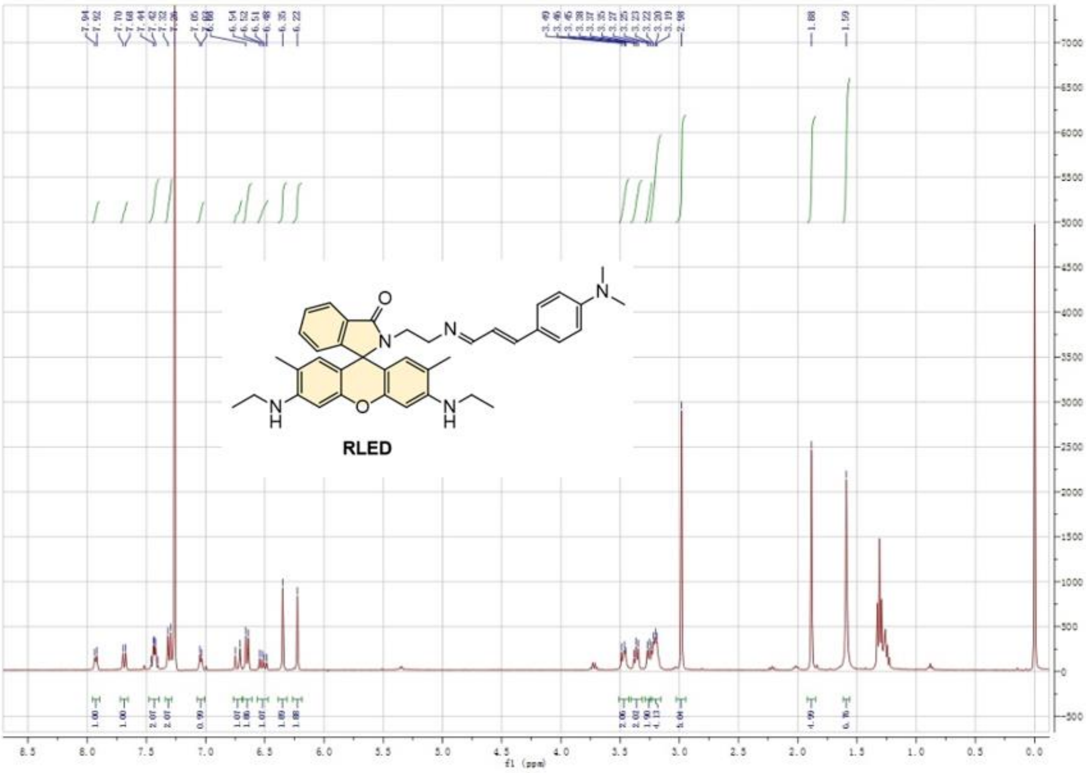

Figure S3. ^{1}H NMR spectra of **RLED.**

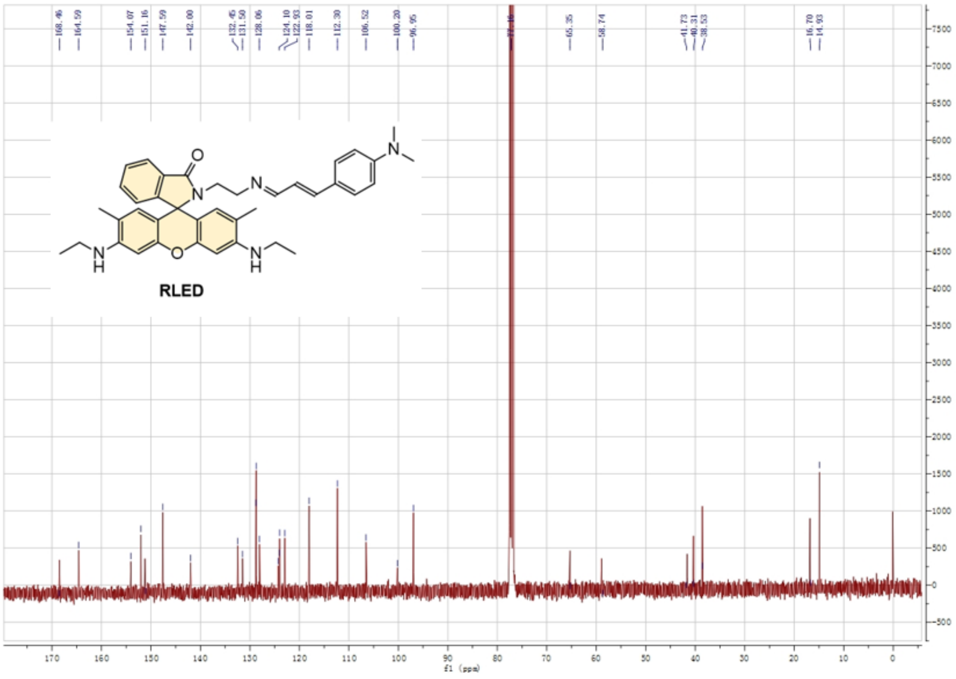

Figure S4. ^{13}C NMR spectra of **RLED.**

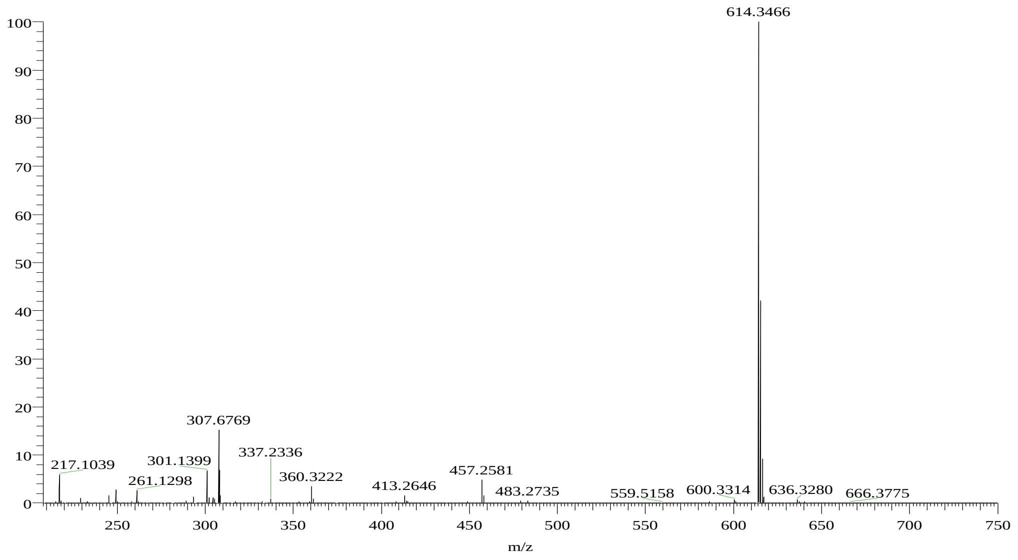

Figure S5. ESI Mass spectrum of **RLED.**

*** Synthesis of Rho-575**

A portion of **RLED** (100 mg, 0.16 mmol) and $Hg(ClO_4)_2$ hydrate (400 mg, 1 mmol) were combined in 100 mL of ethanol. The reaction mixture was refluxed for 2 h. The solvent was removed under reduced pressure, and the residue was dissolved in 100 mL of CH_2Cl_2 and washed with water for 3 times. The organic layer was dried over $MgSO_4$ and the solvent was evaporated under reduced pressure. The crude product was purified by chromatograph to give 16.5 mg of **Rho-575** as red solid in 25% yield. ^{1}H-NMR (DMSO) δ 13.03 (s, 1H), 8.25-8.23 (d, 1H), 7.88-7.85 (t, 1H), 7.83-7.79 (t, 1H), 7.66 (t, 2H), 7.43-7.42 (d, 1H), 6.92 (s, 2H), 6.81 (s, 2H), 3.52-3.45 (t, 4H), 2.10 (s, 6H), 1.28-1.25 (t, 6H). ^{13}C-NMR (DMSO) δ: 166.25, 156.62, 155.65, 132.68, 130.91, 130.73, 130.17, 130.09, 128.52, 125.19, 112.81, 93.55, 37.90, 17.37, 13.59 ppm. ESI-MS ($M+H^+$): m/z = 415.1996.

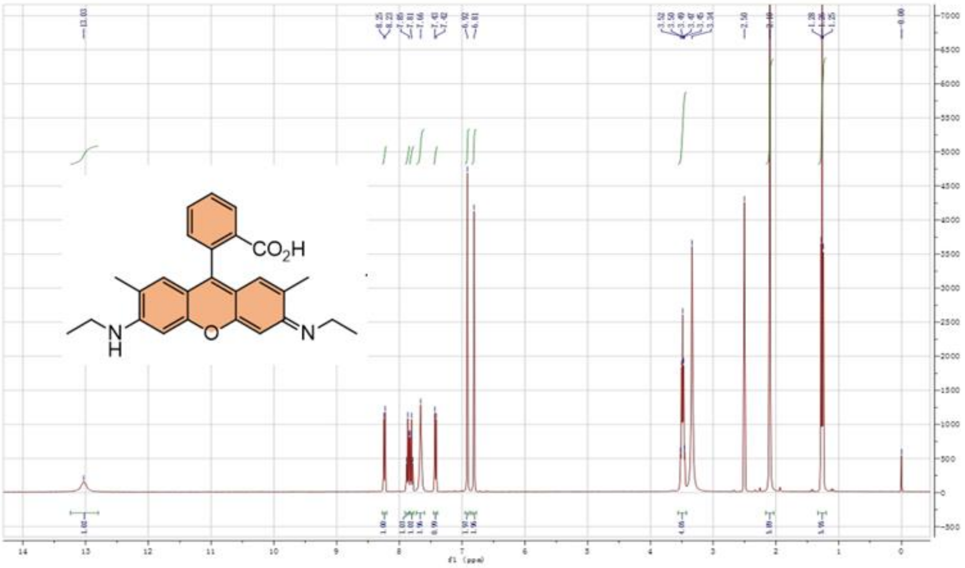

Figure S6. ^{1}H NMR spectra of **Rho-575.**

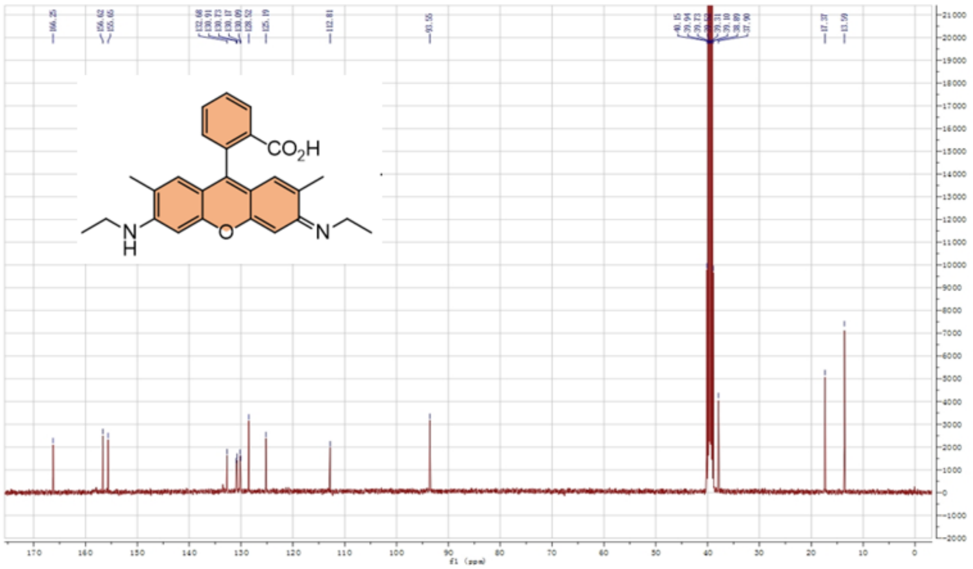

Figure S7. ^{13}C NMR spectra of **Rho-575.**

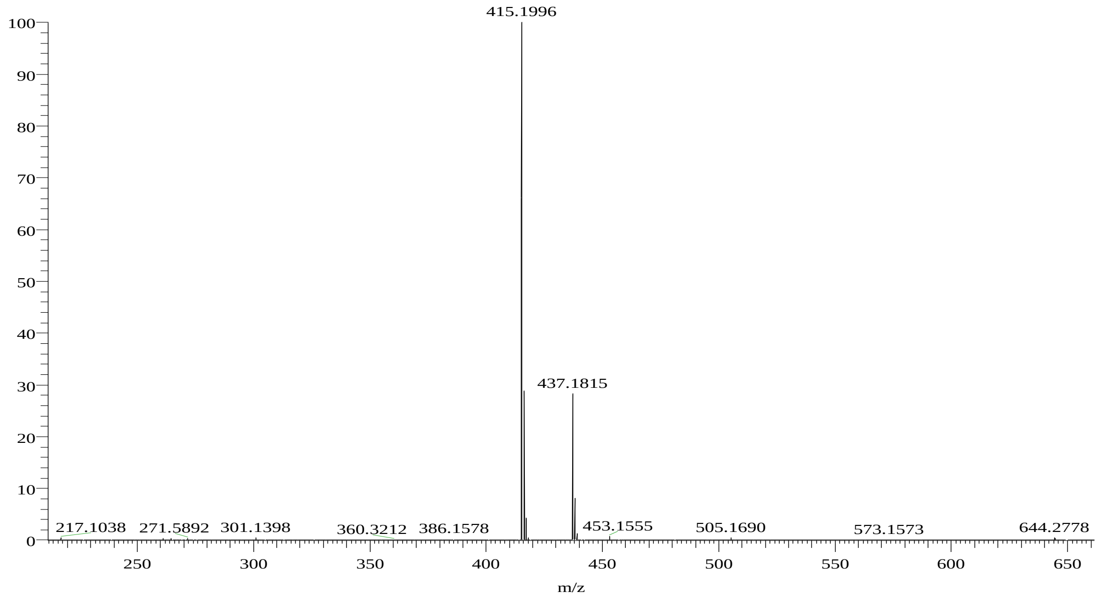

Figure S8. ESI Mass spectrum of **Rho-575.**

2. The variation of absorbance and fluorescence intensity of RLED vs the concentration of Hg^{2+} ions (for calculation of **the detection and quantitation limits**)

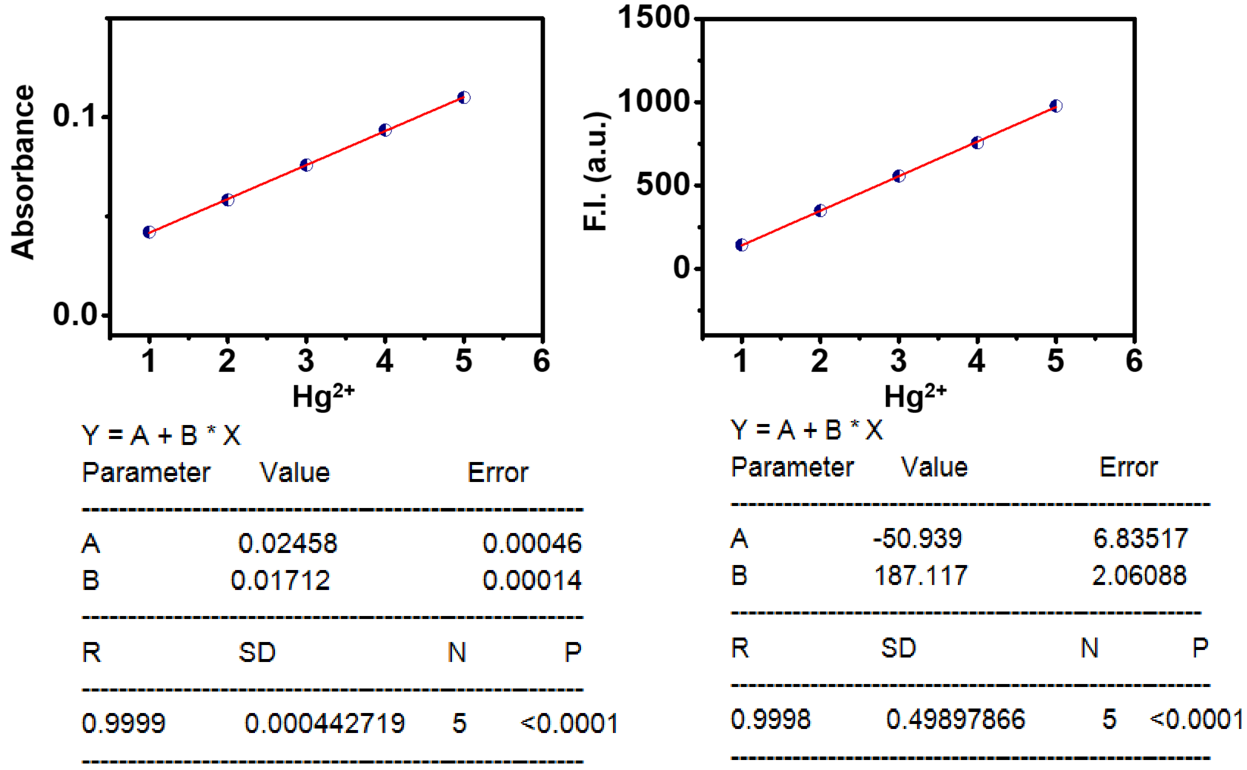

LOD= 3*SD/B = 3*0.000442719/0.01712=0.08 LOD= 3*SD/B = 3*0.49897866/187.117=0.008

LOQ= 10*SD/B = 3*0.000442719/0.01712=0.2 LOQ= 10*SD/B = 10*0.49897866/187.117=0.027

Figure S9. (a) Absorbance at 530 nm *vs* the concentration of Hg^{2+} ions and their linear fitting curve. (b) Emission intensity at 560 nm *vs* the concentration of Hg^{2+} ions and their linear fitting curve. All spectra were recorded at 30 mins after Hg^{2+} addition; **RLED** (10 μM) in MeOH/HEPES (pH 7.4, 1/9, v/v) at 25°C.

[Ref] Miller JC and Miller JN. Statistics for analytical chemistry. second ed. Chichester, England: Ellis Horwood Limited; 1998.

3. Table S1. XYZ coordinates for calculated optimized geometry of RLED

C	-3.59118	3.662711	1.271114
C	-3.45925	2.439656	1.960512
C	-3.24906	1.248893	1.248574
C	-3.16526	1.216218	-0.15144
C	-3.30209	2.45453	-0.82314
C	-3.51107	3.66661	-0.16337
C	-2.8505	-1.27325	0.015536
C	-2.94737	-1.14085	1.408814
C	-2.87046	-2.24219	2.274922

H	-2.94801	-2.05669	3.339572
C	-2.69163	-3.54084	1.755466
C	-2.59704	-3.71515	0.332489
C	-2.67784	-2.58627	-0.48441
H	-3.51008	2.382514	3.041274
H	-3.23763	2.46032	-1.90913
H	-2.60177	-2.7201	-1.5615
O	-3.13281	0.097599	2.043228
C	-2.90125	-0.07305	-0.93351
C	-3.91317	-0.27957	-2.07309
C	-3.26643	-0.25894	-3.31853
C	-5.29722	-0.47115	-1.99483
C	-3.96767	-0.42434	-4.52043
C	-6.01717	-0.63938	-3.19636
H	-5.80657	-0.49057	-1.0349
C	-5.36115	-0.61639	-4.44954
H	-3.43863	-0.40256	-5.46921
H	-7.09402	-0.78912	-3.16077
H	-5.93999	-0.74846	-5.36058
C	-1.80552	-0.04038	-3.10589
O	-0.90104	0.055669	-3.97748
N	-1.62266	0.046723	-1.73603
C	-3.6476	4.966153	-0.93383
H	-4.62062	5.45075	-0.75574
H	-2.86354	5.69037	-0.66175
H	-3.56506	4.790055	-2.01104
C	-2.40866	-5.09971	-0.25807
H	-1.47628	-5.57245	0.089227
H	-3.2384	-5.77501	0.003627
H	-2.35984	-5.05292	-1.35065
N	-2.60921	-4.6475	2.594616
H	-2.44668	-5.5545	2.180767
N	-3.79686	4.856484	1.955655
H	-3.87042	5.711239	1.422558
C	-2.66641	-4.57111	4.055053
H	-1.86545	-3.91442	4.433292
H	-3.62215	-4.12285	4.37177
C	-2.52254	-5.96902	4.676664
H	-1.56142	-6.42471	4.403326
H	-2.56632	-5.90636	5.770091
H	-3.33023	-6.63413	4.343246
C	-3.87693	4.958921	3.413291
H	-4.70409	4.335901	3.791236
H	-2.95194	4.572401	3.872193
C	-4.09369	6.418069	3.843169
H	-5.02858	6.817359	3.427676
H	-4.15276	6.488964	4.935391

H	-3.26432	7.055316	3.50822
C	-0.31577	0.317429	-1.13309
H	0.182488	1.107861	-1.70807
H	-0.46416	0.682015	-0.11176
C	0.605944	-0.92848	-1.10433
H	0.114732	-1.7098	-0.51095
H	0.744599	-1.29678	-2.13583
N	1.867458	-0.57048	-0.43869
C	2.981041	-0.67026	-1.10287
H	3.003475	-1.02384	-2.14917
C	4.266038	-0.32458	-0.5065
H	4.223653	0.026503	0.522572
C	5.444927	-0.43422	-1.1856
H	5.396914	-0.7958	-2.21615
C	6.790312	-0.1229	-0.69782
C	7.905243	-0.30938	-1.55272
C	7.060101	0.364973	0.607746
C	9.213927	-0.0315	-1.1443
H	7.738957	-0.68183	-2.56277
C	8.359111	0.648923	1.033783
H	6.239563	0.526426	1.302612
C	9.479306	0.458145	0.167756
H	10.02665	-0.19498	-1.84361
H	8.507321	1.020805	2.041984
N	10.77851	0.74026	0.590917
C	11.02014	1.237844	1.948849
H	10.67933	0.52305	2.7137
H	12.09238	1.396377	2.093053
H	10.51034	2.196555	2.130524
C	11.90867	0.534316	-0.31995
H	11.81947	1.15089	-1.22762
H	12.83829	0.812464	0.183998
H	11.99587	-0.51778	-0.63276

4. Table S2. XYZ coordinates for calculated optimized geometry of C_1

C	-3.29058	3.817757	-1.82839
C	-3.59882	3.510798	-0.48256
C	-3.62032	2.179344	-0.05042
C	-3.3342	1.102945	-0.90987
C	-3.02272	1.430004	-2.25594
C	-2.99202	2.738362	-2.73797
C	-3.95756	-0.44725	0.964699
C	-4.22588	0.689789	1.749841
C	-4.78171	0.604549	3.030504
H	-4.97787	1.52628	3.564248
C	-5.10057	-0.65807	3.583714
C	-4.8406	-1.8458	2.806357

C	-4.28362	-1.7056	1.536963
H	-3.85509	4.282121	0.23334
H	-2.82262	0.621415	-2.95668
H	-4.10628	-2.6065	0.952925
O	-3.95707	1.992616	1.297885
C	-3.32005	-0.33256	-0.40774
C	-3.8464	-1.34706	-1.42298
C	-2.83463	-2.23523	-1.83942
C	-5.13884	-1.46011	-1.94307
C	-3.07267	-3.25377	-2.77749
C	-5.39356	-2.48163	-2.88228
H	-5.92746	-0.78165	-1.63224
C	-4.37333	-3.37069	-3.29802
H	-2.27949	-3.92652	-3.09055
H	-6.39213	-2.59009	-3.29695
H	-4.60249	-4.14566	-4.02369
C	-1.59076	-1.87669	-1.14376
O	-0.43246	-2.46719	-1.28903
N	-1.82355	-0.83074	-0.33194
C	-2.6837	3.034293	-4.19267
H	-2.4743	2.113844	-4.74713
H	-3.52928	3.526347	-4.69643
H	-1.80903	3.693017	-4.30418
C	-5.19097	-3.21061	3.365541
H	-6.26761	-3.30182	3.573859
H	-4.93358	-4.00515	2.657989
H	-4.65609	-3.4213	4.303963
N	-5.66081	-0.76697	4.839115
H	-5.9077	-1.68583	5.181922
N	-3.28391	5.122212	-2.28046
H	-3.11855	5.294127	-3.26328
C	-6.00304	0.377195	5.697821
H	-5.09838	0.96998	5.903539
H	-6.71321	1.037373	5.175808
C	-6.61866	-0.10512	7.019588
H	-5.91801	-0.74041	7.576637
H	-6.8701	0.751802	7.653721
H	-7.54221	-0.672	6.844665
C	-3.63992	6.286051	-1.45415
H	-4.65703	6.16177	-1.05039
H	-2.95553	6.353436	-0.59451
C	-3.56375	7.578761	-2.27987
H	-4.26308	7.552365	-3.12559
H	-3.8287	8.440154	-1.65739
H	-2.55062	7.744322	-2.66849
C	-0.87244	-0.22946	0.606715
H	-1.47475	0.284784	1.360925

H	-0.32264	-1.01986	1.13573
C	0.13845	0.779246	-0.02207
H	-0.19979	1.049159	-1.0297
H	0.139676	1.701362	0.573269
N	1.535878	0.256605	-0.07722
C	2.547826	0.964858	0.438776
H	2.27939	1.915665	0.910305
C	3.914963	0.617696	0.44419
H	4.224936	-0.31864	-0.02402
C	4.865106	1.457986	1.037572
H	4.477002	2.378209	1.480975
C	6.272634	1.279175	1.144481
C	7.053189	2.286456	1.803042
C	6.981905	0.142021	0.63313
C	8.4249	2.180857	1.945867
H	6.548754	3.163333	2.204755
C	8.352472	0.018793	0.766695
H	6.439059	-0.65165	0.125248
C	9.131369	1.037337	1.430712
H	8.964383	2.970713	2.45382
H	8.845434	-0.858	0.363939
N	10.4861	0.918782	1.564282
C	11.19938	-0.26625	1.040383
H	11.08629	-0.35036	-0.04822
H	12.26321	-0.17345	1.262699
H	10.83498	-1.1891	1.509521
C	11.2724	1.975924	2.238714
H	10.96139	2.096674	3.284245
H	12.32807	1.701546	2.228825
H	11.1665	2.938536	1.722806
Hg	1.772641	-1.6955	-1.08391
O	1.549073	-4.12055	-1.03469
H	0.56272	-4.07527	-1.13037
H	1.852133	-4.86418	-0.48043
O	2.915168	-2.63272	-2.88435
H	2.751554	-3.59846	-2.87179
H	3.36354	-2.31509	-3.68998

5. Table S3. XYZ coordinates for calculated optimized geometry of C_2

C	3.733159	4.645108	0.359557
C	4.469943	3.740779	-0.4493
C	4.411274	2.379951	-0.18
C	3.63136	1.827167	0.885132
C	2.875446	2.764199	1.665481
C	2.905418	4.127694	1.440371
C	4.464864	-0.40172	0.294391
C	5.221917	0.184239	-0.76947

C	6.055287	-0.55122	-1.60184
H	6.60291	-0.03346	-2.37912
C	6.188849	-1.94955	-1.39836
C	5.446316	-2.58954	-0.32132
C	4.621514	-1.81635	0.474274
H	5.093681	4.08361	-1.26511
H	2.25846	2.37774	2.470326
H	4.077732	-2.29193	1.2847
O	5.161387	1.5519	-1.00521
C	3.63316	0.421535	1.106707
C	2.929294	-0.15494	2.300572
C	1.717805	-0.8974	2.239577
C	3.555727	0.033717	3.552853
C	1.175379	-1.42689	3.433099
C	3.007666	-0.50194	4.731535
H	4.485698	0.594185	3.602056
C	1.809212	-1.2354	4.671227
H	0.239182	-1.97315	3.384799
H	3.511581	-0.34726	5.681415
H	1.372899	-1.64622	5.577458
C	0.980289	-1.11283	0.95132
O	0.088379	-2.15296	0.951969
N	1.222381	-0.31597	-0.05021
C	2.105929	5.08615	2.299765
H	1.526254	4.545049	3.052987
H	2.756327	5.790727	2.838886
H	1.39525	5.678062	1.704168
C	5.601145	-4.07808	-0.08389
H	6.641504	-4.34793	0.149754
H	4.988563	-4.40461	0.761341
H	5.291905	-4.66848	-0.9591
N	7.011218	-2.70134	-2.18894
H	7.093289	-3.69282	-1.99813
N	3.789699	5.992658	0.141028
H	3.2613	6.604539	0.751469
C	7.834709	-2.17447	-3.29534
H	7.182149	-1.69231	-4.03783
H	8.519332	-1.40582	-2.90784
C	8.636384	-3.30498	-3.95574
H	7.974746	-4.07167	-4.37856
H	9.244202	-2.90337	-4.77318
H	9.317367	-3.78273	-3.24
C	4.608914	6.64811	-0.89773
H	5.664698	6.374623	-0.75626
H	4.303074	6.283418	-1.88928
C	4.448429	8.173328	-0.82676
H	4.78052	8.565411	0.14283

H	5.05714	8.649572	-1.60239
H	3.405668	8.474174	-0.9901
C	0.661649	-0.5212	-1.39433
H	1.465082	-0.28673	-2.10455
H	0.368551	-1.57029	-1.60415
C	-0.50922	0.420265	-1.7474
H	-0.28517	1.415597	-1.34091
H	-0.5739	0.496951	-2.84255
N	-1.84244	-0.03467	-1.23048
C	-2.91602	0.730852	-1.43872
H	-2.74697	1.675816	-1.96633
C	-4.24571	0.448966	-1.05084
H	-4.44976	-0.48209	-0.51841
C	-5.28641	1.333002	-1.34428
H	-5.00632	2.24083	-1.88428
C	-6.67382	1.210024	-1.03795
C	-7.57313	2.243959	-1.45604
C	-7.24525	0.103358	-0.32919
C	-8.93232	2.18913	-1.19855
H	-7.17486	3.100789	-1.99654
C	-8.60073	0.031493	-0.06148
H	-6.60772	-0.70739	0.01513
C	-9.50092	1.07436	-0.48933
H	-9.56599	2.99864	-1.53965
H	-8.98615	-0.82504	0.478895
N	-10.8426	1.003576	-0.23002
C	-11.413	-0.14832	0.499835
H	-10.9902	-0.22989	1.509704
H	-12.4915	-0.01528	0.596066
H	-11.2344	-1.08867	-0.03759
C	-11.753	2.08025	-0.67614
H	-11.7356	2.188579	-1.76824
H	-12.7734	1.83685	-0.37689
H	-11.4852	3.042195	-0.22032
Hg	-1.89531	-1.97019	-0.18051
O	-2.54519	-3.9317	0.619147
O	-0.55496	-4.37999	1.883383
H	-1.74688	-4.32104	1.243912
H	-3.31125	-4.49852	0.430037
H	-0.02252	-3.53656	1.660377
H	-0.1198	-5.08358	2.387825

6. Table S4. XYZ coordinates for calculated optimized geometry of C_3

C	-3.42753	4.091877	0.407226
C	-3.68308	3.098232	1.3886
C	-3.62054	1.747124	1.045185
C	-3.2999	1.312147	-0.25802

C	-3.04259	2.321341	-1.22484
C	-3.09626	3.68396	-0.94056
C	-3.84736	-1.0214	0.481509
C	-4.14563	-0.49492	1.75675
C	-4.69354	-1.27177	2.776993
H	-4.91375	-0.79644	3.724851
C	-4.97061	-2.64553	2.54802
C	-4.67848	-3.22076	1.252225
C	-4.13138	-2.39699	0.271905
H	-3.95888	3.357606	2.403408
H	-2.81365	2.016537	-2.24318
H	-3.91898	-2.82812	-0.70285
O	-3.91259	0.850201	2.083241
C	-3.19394	-0.16863	-0.60078
C	-3.68703	-0.50584	-2.01071
C	-2.64578	-1.03589	-2.79431
C	-4.97119	-0.36237	-2.5444
C	-2.84244	-1.44252	-4.12231
C	-5.18419	-0.76409	-3.8812
H	-5.78603	0.044422	-1.95249
C	-4.13407	-1.29942	-4.66347
H	-2.02512	-1.85731	-4.70468
H	-6.17366	-0.66337	-4.31894
H	-4.33115	-1.60367	-5.6873
C	-1.41654	-1.07287	-1.97941
O	-0.26465	-1.50602	-2.34882
N	-1.70751	-0.57197	-0.74443
C	-2.84114	4.724109	-2.01269
H	-3.72331	5.358551	-2.18439
H	-2.00161	5.387221	-1.75564
H	-2.59952	4.251089	-2.96902
C	-4.97406	-4.68113	0.979491
H	-4.41945	-5.34752	1.656805
H	-6.04434	-4.91059	1.090125
H	-4.69333	-4.95374	-0.04179
N	-5.51807	-3.42956	3.533382
H	-5.71752	-4.40044	3.320501
N	-3.50592	5.42826	0.717656
H	-3.34668	6.104212	-0.02045
C	-5.878	-2.96589	4.885921
H	-4.98447	-2.55626	5.380174
H	-6.61369	-2.1511	4.809696
C	-6.45634	-4.11948	5.717577
H	-5.72768	-4.93099	5.83823
H	-6.72343	-3.76126	6.717189
H	-7.36548	-4.52742	5.258016
C	-3.87411	5.970779	2.038243

H	-4.87057	5.600229	2.322244
H	-3.1618	5.607623	2.794109
C	-3.87445	7.505989	2.011859
H	-4.60182	7.893076	1.28715
H	-4.1502	7.896092	2.997113
H	-2.88247	7.903009	1.761814
C	-0.73791	-0.47506	0.352087
H	-1.30744	-0.36737	1.278663
H	-0.17876	-1.41558	0.42155
C	0.277721	0.709176	0.230976
H	-0.04327	1.376632	-0.57772
H	0.264415	1.304603	1.156286
N	1.660775	0.23344	-0.03308
C	2.652208	0.611147	0.756671
H	2.404669	1.21039	1.643474
C	4.034801	0.306095	0.566785
H	4.332182	-0.14733	-0.37822
C	4.981511	0.613636	1.53463
H	4.613262	1.088897	2.446744
C	6.395321	0.377597	1.49143
C	7.199513	0.766381	2.610403
C	7.06996	-0.23293	0.384537
C	8.570145	0.570333	2.635485
H	6.719171	1.232491	3.468379
C	8.438204	-0.43726	0.390725
H	6.505644	-0.5483	-0.48892
C	9.243997	-0.04231	1.520647
H	9.131543	0.885528	3.506558
H	8.904342	-0.90175	-0.46993
N	10.59745	-0.24292	1.530541
C	11.28039	-0.8739	0.379532
H	11.14381	-0.28318	-0.53513
H	12.34952	-0.9361	0.585281
H	10.90723	-1.89131	0.206356
C	11.4128	0.165084	2.696461
H	11.09593	-0.36267	3.604854
H	12.45863	-0.08037	2.508683
H	11.34166	1.246289	2.869908
Hg	2.004825	-0.80601	-2.19258

7. Table S5. XYZ coordinates for calculated optimized geometry of C_4

C	4.600965	4.157527	-0.20298
C	4.967591	3.066685	-1.03509
C	4.68209	1.770795	-0.62783
C	4.024444	1.466514	0.607299

C	3.639752	2.591946	1.410896
C	3.904845	3.899632	1.050531
C	4.241837	-0.92287	0.096606
C	4.886048	-0.58131	-1.13575
C	5.370705	-1.52986	-2.02645
H	5.858764	-1.19207	-2.93197
C	5.243705	-2.90952	-1.71587
C	4.601134	-3.30644	-0.47039
C	4.128709	-2.32526	0.381007
H	5.481428	3.215286	-1.9764
H	3.120814	2.39941	2.344443
H	3.665687	-2.6242	1.316208
O	5.072593	0.750414	-1.48614
C	3.784186	0.110681	0.964272
C	3.235801	-0.23131	2.317451
C	1.938794	-0.76755	2.552193
C	4.111719	-0.06436	3.412975
C	1.571264	-1.14611	3.864144
C	3.730979	-0.43441	4.715476
H	5.105697	0.339741	3.24059
C	2.456058	-0.98355	4.941391
H	0.58753	-1.57315	4.022293
H	4.427017	-0.30312	5.539169
H	2.156546	-1.28014	5.942426
C	0.936278	-0.92457	1.450474
O	0.036679	-1.87745	1.763959
N	1.05567	-0.21313	0.367713
C	3.494337	5.059096	1.93546
H	2.974845	4.703008	2.829496
H	4.363637	5.639286	2.278585
H	2.814685	5.751471	1.417273
C	4.479725	-4.77603	-0.12146
H	5.464944	-5.25695	-0.03287
H	3.970522	-4.9091	0.837109
H	3.903843	-5.33191	-0.87604
N	5.72581	-3.869	-2.55966
H	5.635888	-4.84121	-2.28897
N	4.890594	5.443427	-0.55955
H	4.625739	6.191759	0.070115
C	6.423506	-3.604	-3.83369
H	5.762685	-3.02871	-4.49843
H	7.315432	-2.98986	-3.64145
C	6.829691	-4.92148	-4.50945
H	5.953345	-5.54149	-4.73717
H	7.344317	-4.71366	-5.45334
H	7.516268	-5.49962	-3.87818
C	5.610567	5.840672	-1.78599

H	6.596095	5.353019	-1.80448
H	5.051652	5.491458	-2.66646
C	5.779502	7.365617	-1.84165
H	6.361282	7.734398	-0.9875
H	6.315232	7.648713	-2.75368
H	4.808386	7.876636	-1.85388
C	0.331163	-0.39284	-0.90893
H	1.084904	-0.23079	-1.68931
H	0.049198	-1.42117	-1.07989
C	0.804724	0.630757	-1.19946
H	0.537321	1.586818	-0.73022
H	0.862677	0.779964	-2.28635
N	2.161599	0.193777	-0.72085
C	3.280281	0.732451	-1.21707
H	3.147543	1.490392	-1.99701
C	4.601935	0.423388	-0.84201
H	4.76167	-0.32644	-0.0652
C	5.693722	1.059322	-1.44706
H	5.452259	1.797639	-2.21557
C	7.080731	0.873529	-1.19426
C	8.034084	1.642686	-1.94102
C	7.606208	-0.04654	-0.22643
C	9.39757	1.513943	-1.74925
H	7.671839	2.35109	-2.68375
C	8.965565	-0.18898	-0.0212
H	6.928319	-0.6538	0.368245
C	9.919019	0.587915	-0.77781
H	0.072253	2.120245	-2.34104
H	9.31527	-0.89656	0.721105
N	1.263371	0.448383	-0.57852
C	1.787006	-0.50684	0.422309
H	1.439358	-0.25533	1.432505
H	2.877051	-0.46757	0.421448
H	1.484578	-1.53515	0.186575
C	2.229069	1.252608	-1.36006
H	2.132479	1.054141	-2.43498
H	3.243814	0.990796	-1.05755
H	2.086903	2.326195	-1.18247
Hg	1.898182	-1.34836	0.821524

8. Table S6. XYZ coordinates for calculated optimized geometry of Rho-575

C	-3.65443	-0.0005	-0.31314
C	-3.604	-1.4298	-0.15022
C	-2.3469	-2.07335	-0.06812
C	-1.17636	-1.31746	-0.1398

C	-1.18249	0.091126	-0.29618
C	-2.45822	0.711977	-0.38047
O	0.022188	-2.02474	-0.05237
C	1.255732	-1.35445	-0.11882
C	1.275002	0.093175	-0.27818
C	0.081572	0.800468	-0.35215
C	2.388193	-2.1121	-0.02823
C	3.715205	-1.49548	-0.08422
C	3.756831	-0.01742	-0.24081
C	2.593977	0.701318	-0.32854
N	4.870033	-2.1315	-0.00573
C	-4.8456	-3.60962	0.099621
C	-6.30522	-4.08513	0.158717
C	4.884787	-3.59817	0.153996
C	6.334593	-4.099	0.22756
C	0.079366	2.2864	-0.56392
C	-0.03408	3.242835	0.483826
C	-0.02993	4.626553	0.184949
C	0.079862	5.073986	-1.13926
C	0.193227	4.134063	-2.18128
C	0.193214	2.758805	-1.88966
C	-4.99289	0.707613	-0.40311
C	5.109896	0.650177	-0.29407
C	-0.14514	2.810326	1.904325
O	-0.20556	1.645377	2.33018
O	-0.18048	3.89051	2.789632
H	-2.25889	-3.14622	0.053269
H	-2.50244	1.791188	-0.5015
H	2.286233	-3.18525	0.090728
H	2.646769	1.781074	-0.4383
H	-4.32559	-4.11277	-0.73127
H	-4.32408	-3.89845	1.026123
H	-6.34535	-5.17234	0.290587
H	-6.83736	-3.62384	1.001257
H	-6.83971	-3.83834	-0.7683
H	4.342887	-3.90007	1.069377
H	4.365637	-4.09162	-0.68858
H	6.372777	-5.19074	0.348315
H	6.87686	-3.82752	-0.6863
H	6.853819	-3.63362	1.074104
H	-0.11011	5.333663	1.002624
H	0.081622	6.138795	-1.35627
H	0.28365	4.467196	-3.21227
H	0.2854	2.033797	-2.69355
H	-5.59835	0.558679	0.504662
H	-4.85486	1.786531	-0.5258
H	-5.58878	0.356111	-1.25994

H	5.709121	0.242062	-1.11708
H	5.013739	1.735414	-0.41864
H	5.679026	0.440389	0.619917
N	-4.78335	-2.15599	-0.07694
H	-5.66032	-1.6573	-0.13435
H	-0.24489	3.558329	3.713324

9. Table S7. XYZ coordinates for calculated optimized geometry of ED-DACA-Hg

C	-4.41204	1.778049	0.63186
H	-5.18965	2.55086	0.584712
H	-4.04865	1.726085	1.66367
C	-3.26883	2.218427	-0.32403
H	-3.66454	2.261467	-1.34912
H	-2.97498	3.236322	-0.0334
N	-2.10436	1.303601	-0.3337
C	-0.88662	1.779412	-0.11624
H	-0.77291	2.863218	0.011885
C	0.313495	1.005832	-0.06617
H	0.226935	-0.0793	-0.02504
C	1.559355	1.629205	-0.04451
H	1.560584	2.72047	-0.06447
C	2.848783	1.012403	-0.01504
C	4.014645	1.855195	-0.01267
C	3.062329	-0.41083	0.006791
C	5.29245	1.335501	0.011665
H	3.884542	2.934696	-0.02956
C	4.331408	-0.94946	0.029401
H	2.213617	-1.08837	0.003458
C	5.50386	-0.09572	0.03175
H	6.137661	2.012549	0.014544
H	4.447257	-2.02639	0.045163
N	6.756502	-0.62225	0.052113
C	6.97372	-2.09205	0.066346
H	6.542152	-2.55869	-0.82705
H	8.043826	-2.29648	0.074769
H	6.530853	-2.54345	0.962129
C	7.956988	0.254775	0.05834
H	7.961345	0.902393	0.942977
H	8.85342	-0.36401	0.082639
H	7.990602	0.875294	-0.84503
N	-4.97776	0.440476	0.269161
H	-5.53951	0.034227	1.022316
H	-5.53536	0.464779	-0.59057
Hg	-2.92656	-0.94254	-0.03878

10. Table S8. The calculated excitation energy (*E*), wavelength (*λ*), and oscillator strength (*f*) for low-laying singlet state of C_1 at the B3LYP/LanL2DZ level (in water).

State	MO	*E* (eV)	λ (nm)	*f*	Percentage contribution (%)
$S_0 \to S_1$	177→180	2.70	458.83	0.0006	2.07
	178→180				2.26
	179→180				95.32
$S_0 \to S_2$	177→180	2.85	434.80	0.0001	97.05
$S_0 \to S_3$	178→180	2.99	414.41	1.6022	96.09
	179→180				2.33
$S_0 \to S_4$	178→181	3.02	409.96	0.0008	2.18
	179→181				96.85
$S_0 \to S_5$	177→181	3.11	398.64	0.0010	97.62
$S_0 \to S_6$	178→181	3.24	382.26	0.0410	95.28
	179→181				2.18

11. Table S9. The calculated excitation energy (*E*), wavelength (*λ*), and oscillator strength (*f*) for low-laying singlet state of C_2 at the B3LYP/LanL2DZ level (in water).

State	MO	*E* (eV)	λ (nm)	*f*	Percentage contribution (%)
$S_0 \to S_1$	179→180	2.30	538.33	0.0030	99.83
$S_0 \to S_2$	178→180	2.74	452.53	0.8299	95.21
$S_0 \to S_3$	177→180	2.89	428.97	0.5591	79.77
	178→180				2.12
	179→181				14.73
$S_0 \to S_4$	176→180	3.01	412.42	1.1385	6.65
	177→180				12.23
	179→181				80.17
$S_0 \to S_5$	176→180	3.07	404.52	0.0182	87.87
	179→181				4.75
$S_0 \to S_6$	178→181	3.13	396.60	0.0011	99.73

12. Table S10. The calculated excitation energy (*E*), wavelength (*λ*), and oscillator strength (*f*) for low-laying singlet state of C_3 at the B3LYP/LanL2DZ level (in water).

State	MO	*E* (eV)	λ (nm)	*f*	Percentage contribution (%)
$S_0 \to S_1$	168→170	2.49	498.08	0.0689	77.33
	168→171				9.83
	169→170				10.17
$S_0 \to S_2$	168→170	2.57	482.56	0.0020	11.23
	169→170				85.99
$S_0 \to S_3$	167→170	2.70	458.54	0.0006	96.09
$S_0 \to S_4$	168→171	2.84	436.20	0.0029	7.44
	169→171				89.53
$S_0 \to S_5$	167→171	2.99	414.65	0.0024	96.04
$S_0 \to S_6$	168→170	3.05	407.18	1.4715	10.08
	168→171				80.60
	169→171				6.16

13. Table S11. The calculated excitation energy (*E*), wavelength (*λ*), and oscillator strength (*f*) for low-laying singlet state of **C_4** at the B3LYP/LanL2DZ level (in water).

State	MO	*E* (eV)	λ (nm)	*f*	Percentage contribution (%)
$S_0 \rightarrow S_1$	169→170	2.30	539.79	0.0103	99.68
$S_0 \rightarrow S_2$	167→170	2.74	453.07	0.8088	5.08
	168→170				92.80
$S_0 \rightarrow S_3$	167→170	2.81	441.85	0.4326	85.61
	168→170				5.10
	169→171				5.93
$S_0 \rightarrow S_4$	167→170	3.00	413.79	1.3455	5.80
	169→171				92.99
$S_0 \rightarrow S_5$	166→170	3.08	402.05	0.0352	97.31
$S_0 \rightarrow S_6$	168→171	3.13	395.92	0.0021	99.73

14. Table S12. The calculated excitation energy (*E*), wavelength (*λ*), and oscillator strength (*f*) for low-laying singlet state of **Rho-575** at the B3LYP/LanL2DZ level (in water).

State	MO	*E* (eV)	λ (nm)	*f*	Percentage contribution (%)
$S_0 \rightarrow S_1$	110→111	2.37	523.43	0.0173	17.1
	110→112				82.59
$S_0 \rightarrow S_2$	110→111	2.70	459.96	0.9333	81.48
	110→112				16.90
$S_0 \rightarrow S_3$	108→111	3.31	375.06	0.0004	94.77
	108→112				3.71
$S_0 \rightarrow S_4$	107→111	3.43	361.08	0.0408	13.83
	109→111				81.66
	110→115				2.88
$S_0 \rightarrow S_5$	110→113	3.60	344.36	0.0090	96.87
$S_0 \rightarrow S_6$	107→111	3.60	344.05	0.0024	5.96
	109→112				89.57

15. Table S13. The calculated excitation energy (*E*), wavelength (*λ*), and oscillator strength (*f*) for low-laying singlet state of **ED-DACA-Hg** at the B3LYP/LanL2DZ level (in water).

State	MO	*E* (eV)	λ (nm)	*f*	Percentage contribution (%)
$S_0 \rightarrow S_1$	64→65	2.61	475.11	0.1441	72.81
	64→66				26.22
$S_0 \rightarrow S_2$	64→65	3.09	401.07	1.2660	26.37

	64→66				72.43
	62→65	4.19	295.86	0.0254	15.24
$S_0 \rightarrow S_3$	62→66				6.64
	64→67	4.32	287.23	0.0219	75.22
	61→65				4.21
$S_0 \rightarrow S_4$	63→65				67.72
	63→66				22.30
	61→65	4.39	282.72	0.0019	78.32
$S_0 \rightarrow S_5$	61→66				11.12
	63→65				6.22
	62→65	4.58	270.63	0.0162	65.11
	62→66				2.24
$S_0 \rightarrow S_6$	63→66				10.35
	64→67				16.09
	64→68				2.34

16. Figure S10. The frontier orbital energy diagram of $\mathbf{C_1}$ (The energy levels are relative, not in proportion).

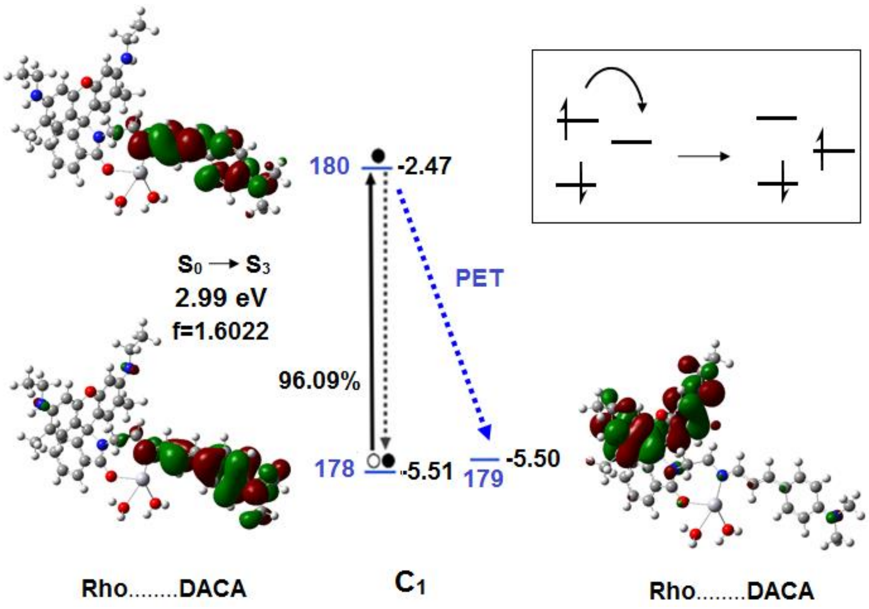

17. Figure S11. The frontier orbital energy diagram of $\mathbf{C_2}$ (The energy levels are relative, not in proportion)

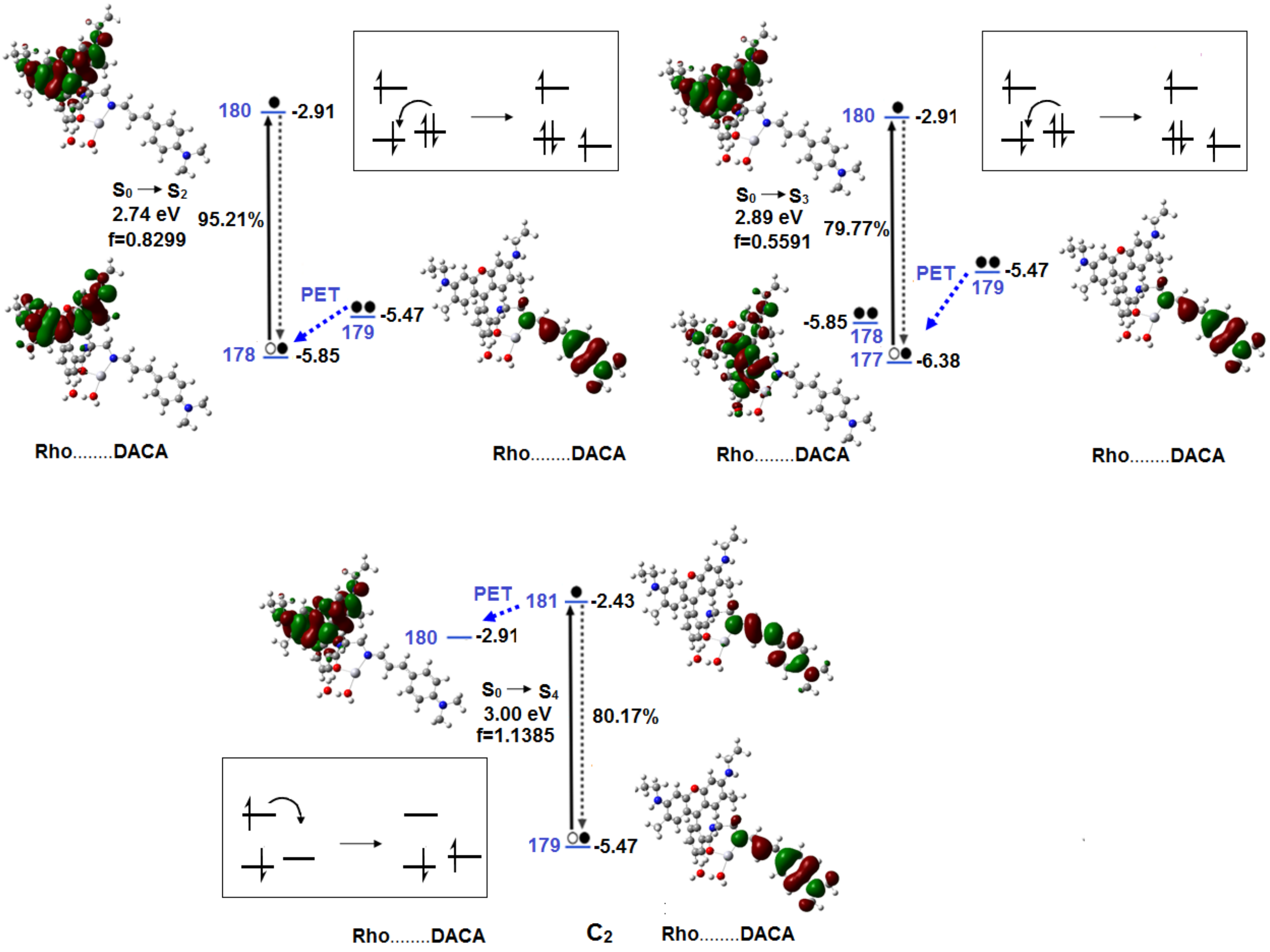

18. Figure S12. The frontier orbital energy diagram of **C_3** (The energy levels are relative, not in proportion)

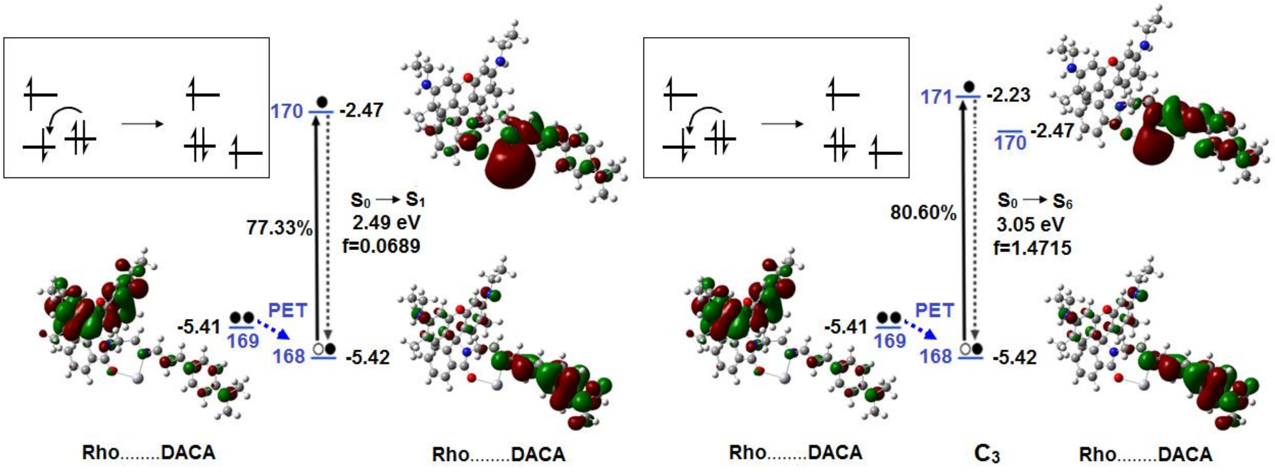

19. Figure S13. The frontier orbital energy diagram of **C_4** (The energy levels are relative, not in proportion)

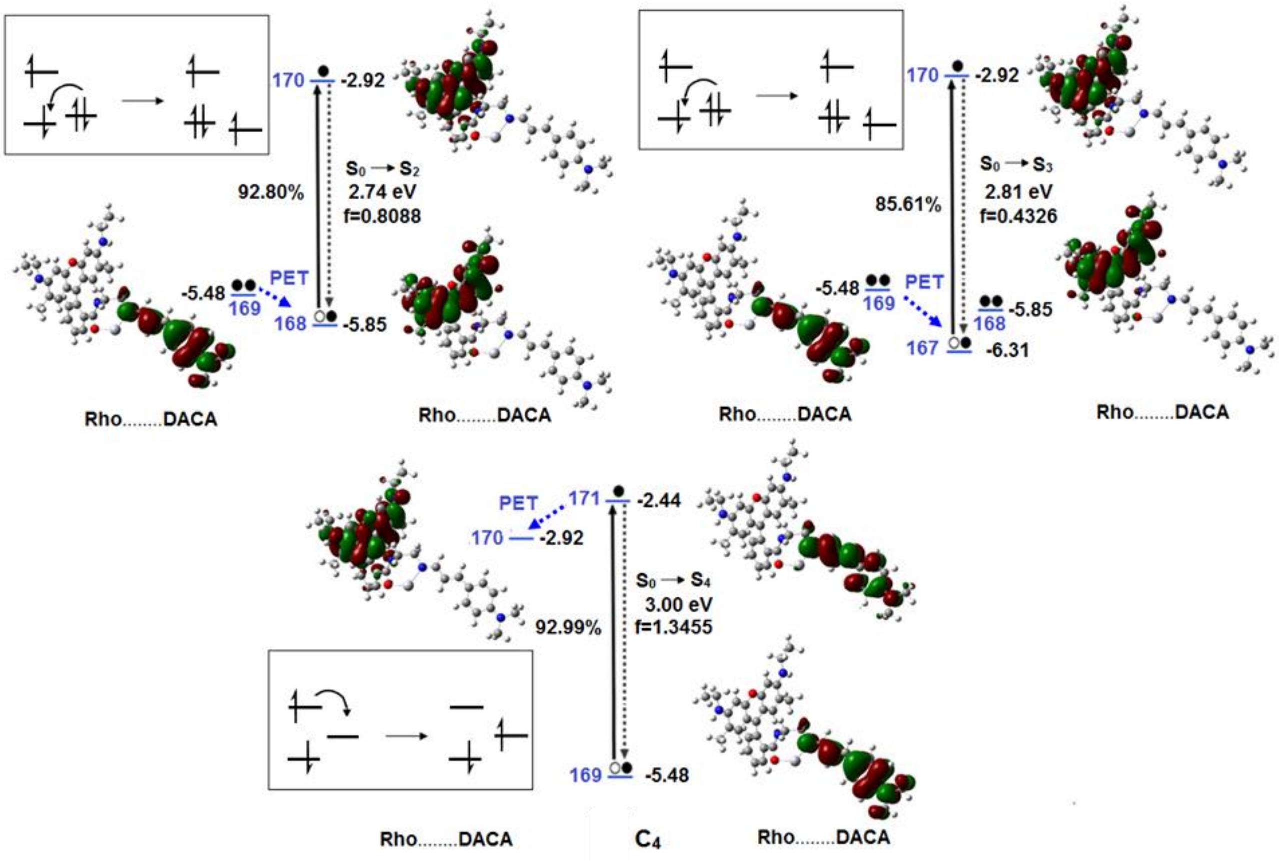

20. Figure S14. The frontier orbital energy diagram of **Rho-575** (The energy levels are relative, not in proportion)

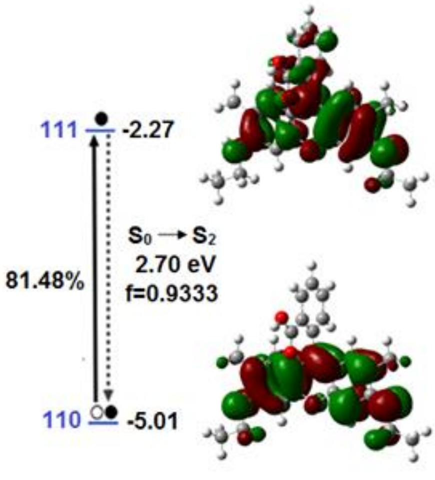

Rho-575

21. Figure S15. The frontier orbital energy diagram of **ED-DACA-Hg** (The energy levels are relative, not in proportion)

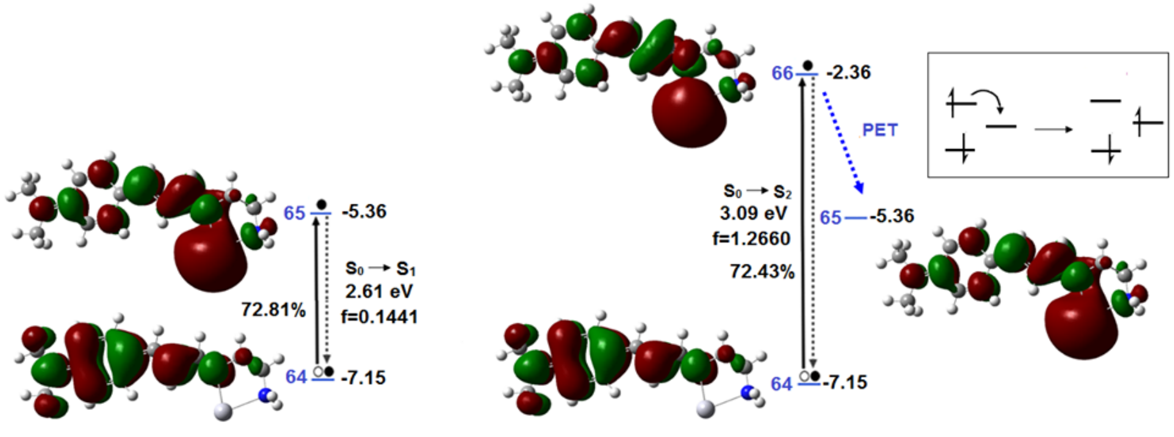

ED-DACA-Hg
